# DMTF1 up-regulation rescues proliferation defect of telomere dysfunctional neural stem cells via the SWI/SNF-E2F axis

**DOI:** 10.1126/sciadv.ady5905

**Published:** 2026-01-02

**Authors:** Yajing Liang, Oleg V. Grinchuk, Nadia Omega Cipta, Yingying Zeng, You Heng Chuah, Jeehyun Yoon, Zi Jian Khong, Hui Ying Chow, Winanto Ng, Chin Tong Ong, Shuo-Chien Ling, Shi-Yan Ng, Yuin-Han Loh, Derrick Sek Tong Ong

**Affiliations:** ^1^ Department of Physiology, Yong Loo Lin School of Medicine, National University of Singapore, Singapore 117593, Singapore.; ^2^ Healthy Longevity Translation Research Program, Yong Loo Lin School of Medicine, National University of Singapore, Singapore 117456, Singapore.; ^3^ NUS Centre for Cancer Research Translation Research Program, Yong Loo Lin School of Medicine, National University of Singapore, Singapore 117599, Singapore.; ^4^ Institute of Molecular and Cell Biology (IMCB), Agency for Science, Technology and Research (A*STAR), Singapore 138673, Singapore.; ^5^ Temasek Life Sciences Laboratory, National University of Singapore, Singapore 117604, Singapore.; ^6^ Department of Biological Sciences, National University of Singapore, Singapore 117543, Singapore.; ^7^ National Neuroscience Institute, Singapore 308433, Singapore.

## Abstract

Impaired neural stem cell (NSC) proliferation/activation is associated with brain aging, but the underlying mechanisms remain poorly understood. Here, we unexpectedly find that DMTF1, a transcription factor that regulates the Arf/p53 axis in cancer, is down-regulated in the NSCs of a premature aging model driven by telomerase deficiency. DMTF1 up-regulation was able to rescue the impaired proliferation of telomere dysfunctional NSCs. Mechanistically, DMTF1 regulates the transcription of Arid2 and Ss18 genes, two subunits of the SWI/SNF complexes that mediate H3K27ac at E2F gene promoters to promote NSC proliferation. Accordingly, Arid2 or Ss18 depletion phenocopies DMTF1 loss in reducing H3K27ac levels, expression of E2F target genes, and NSC proliferation. Thus, our study has identified DMTF1 as a potential therapeutic target to reverse the proliferation defect of aged NSC that is modeled by telomere attrition and unearthed a distinct genetic program controlled by DMTF1 in NSC.

## Teaser

DMTF1 up-regulation promotes proliferation of “aged” neural stem cells by regulating the SWI/SNF-E2F axis.

## INTRODUCTION

Aging is associated with cognitive impairment, loss of neural circuits and synaptic plasticity, increased neuronal vulnerability, and impaired neural stem cell (NSC) proliferation/activation (that contributes to neurogenesis) (*1*–*3*). An important hallmark of aging is the gradual erosion of telomeres (*4*) due to the end replication problem, particularly in cells that express low levels of telomerase. Telomerase is a ribonucleoprotein that comprises the reverse transcriptase TERT and the telomerase RNA (TERC). In mice, telomerase deficiency results in profound NSC loss in the subventricular zone (SVZ) of the brain that compromises neurogenesis, mirroring NSC decline in the aged human brain (*5*–*7*). Notably, defective NSC proliferation can be partially restored in the telomere dysfunctional mice via telomerase reactivation or telomerase gene therapy, although the underlying mechanisms remain poorly understood (*5*, *8*). Given the unique ability of NSCs to self-renew and undergo differentiation into various brain cell types, understanding the mechanisms that contribute to NSC proliferation defect in the telomere dysfunctional mice may guide the development of innovative NSC regenerative medicine. Despite our increased appreciation of the molecular basis of aging-induced NSC impairment, including defects in NSC adhesion and migration, changes in mitochondrial morphology and function, as well as impaired lysosome-autophagy pathway (*9*–*11*), the current lack of effective interventions to restore the function of aged NSCs underscores the urgent need for further research in this area.

DMTF1 (or DMP1) is a transcription factor that harbors both cyclin D binding (CDB) and Myb-like (Myb) domains and thought to act as a tumor suppressor in certain cancer types (*12*). In human lung carcinomas, DMTF1 loss of heterozygosity (LOH) at a frequency of ~35% has been reported, and DMTF1 LOH or null accelerates tumorigenesis in a *K-Ras^LA^* lung cancer mouse model relative to the DMTF1 intact counterpart (*13*). Loss of DMTF1 also promotes tumorigenesis in mouse models for lymphomas [e.g., Eμ-*Myc*–induced B cell lymphomas (*14*)] and breast cancer [e.g., MMTV-neu model (*15*)]. The tumor suppressor activity of DMTF1 may be partially explained by the role of DMTF1 in the transcriptional activation of Arf, leading to p53-dependent cell cycle arrest (*16*) and/or the binding of DMTF1 to p53, which promotes p53 stability and nuclear function (by antagonizing MDM2-mediated ubiquitination of p53) (*17*). Notably, the DMTF1 knockout (KO) mice are smaller in size than their wild-type (WT) littermates at birth and exhibit phenotypes, including seizures and poor mammary gland development (*18*). In 8-week-old male mice, DMTF1 mRNA expression is the highest in the testes, thymus, and brain. Furthermore, DMTF1 null murine embryonic fibroblasts exhibit a growth defect (*18*). Collectively, these observations raise the tantalizing possibility that DMTF1 may have tissue-specific and growth-promoting functions. For normal tissues, DMTF1 has only been investigated in the hematopoietic compartment whereby DMTF1 loss increases hematopoietic stem cell self-renewal and differentiation, which is linked to reduced p21 and Arf levels (*19*). Whether DMTF1 regulates NSC function and its relevance to NSC aging remains unknown.

The SWI/SNF (Brg/Brm-associated factor, BAF) chromatin remodeling complexes are large multisubunit assemblies that regulate nucleosomal structures via adenosine 5′-triphosphate hydrolysis and implicated in carcinogenesis as cancer genome sequencing revealed mutations in SWI/SNF genes in more than 20% of cancers (*20*–*22*). There are at least three broad families of SWI/SNF complexes in mammalian cells that include canonical BAF (cBAF), polybromo-associated BAF (PBAF), and noncanonical BAF (ncBAF). Apart from the common core components (e.g., SMARCC1, SMARCC2, and SMARCD1), SWI/SNF complexes contain either adenosine triphosphatases SMARCA4 or SMARCA2 and other cell type–specific variable subunits (*21*). Of relevance to this study, the Ss18 subunit is found in the cBAF and ncBAF complexes, while Arid2 belongs to the PBAF complex. It is thought that the different subfamilies may have unique DNA binding profiles, and their interactions with specific nuclear proteins might be determined by their different subunits (*21*). Early works demonstrate opposing epigenetic roles of SWI/SNF and Polycomb complexes in development and cancer. For instance, Brg1 (or SMARCA4) mutation leads to H3K27me3 accumulation and silencing of many STAT3 target genes in embryonic stem cells (ESCs) (*23*), and SNF5 (or SMARCB1) loss increases EZH2 expression and hence H3K27me3-mediated repression of Polycomb targets (*24*). More recently, it was shown that the deletion of SMARCC1 or SMARCA4 causes the loss of H3K27ac levels and enhancer activities that resulted in the down-regulation of developmental genes (*25*). Thus, SWI/SNF complexes preferentially target enhancers to provide chromatin accessibility for the binding of transcription factors necessary for gene activation (*21*).

Besides having a prominent role in cancer [for instance, by regulating cell cycle genes such as p21 (*26*) and c-myc (*27*)], the SWI/SNF complexes also exert a profound influence on NSC proliferation and lineage decision. For example, Brg1-deficient NSCs exhibit premature neuronal differentiation and a block in astrocyte and oligodendrocyte differentiation, which is associated with the down-regulation of Sox1, Musashi-1, and Pax6 (*28*). Brg1 also suppresses the expression of Olig2 in NSC, thereby preventing the specification of oligodendrocyte progenitors (*29*). In another instance, the switch from the neural progenitor BAF (npBAF) to neuron-specific nBAF complexes or the loss of Ss18 with the substitution of calcium-responsive element binding protein 1 (CREST) is required for the transition from proliferative NSC to mitotically inactive neurons (*30*). Specifically, the absence of npBAF subunit BAF53a reduces chromatin accessibility at specific neural transcription factor–binding sites, including the pioneer factors SOX2 and ASCL1, leading to Polycomb-mediated repression of cell cycle genes, thereby impeding cell cycle progression and differentiation (*31*). Apart from serving as a classical pioneer factor, ASCL1 can also act as a nonpioneer remodeler to regulate gene regulation in cis by associating with BAF complexes at permissive chromatin to sustain human neural progenitor differentiation (*32*). Recently, it was shown that Snr1 (the Drosophila ortholog of SMARCB1) promotes the transition from neuroepithelial cells to neuroblasts by regulating Notch signaling genes and subsequent neuroblast differentiation by regulating transcription factors Br and Eip93F (*33*). The SWI/SNF complexes may also contribute to the neurodegenerative diseases because of their interaction with various neuroinflammation-related pathways, including transforming growth factor–β/SMAD and hypoxia-inducible factor 1α (*34*). In contrast to our advanced understanding of how SWI/SNF complexes mediate gene transcription, we have very little knowledge of the transcriptional regulation of SWI/SNF genes in the NSC.

In this study, we made an unexpected finding that DMTF1 is down-regulated in telomere dysfunctional NSCs and that DMTF1 up-regulation is sufficient to rescue the proliferation defect of such NSCs in vitro. Using multi-omic analysis, we identified Arid2 and Ss18 as bona fide DMTF1 gene targets that can account for DMTF1’s role in promoting NSC proliferation. In DMTF1-expressing NSCs, we showed that Arid2 and Ss18 were localized to E2F-targeted gene promoters where they mediated H3K27ac levels for gene activation and expression. Thus, DMTF1 represents a potential therapeutic target to restore NSC function during brain aging.

## RESULTS

### DMTF1 up-regulation rescues proliferation defect of telomere dysfunctional NSC

**Fig. 1.**
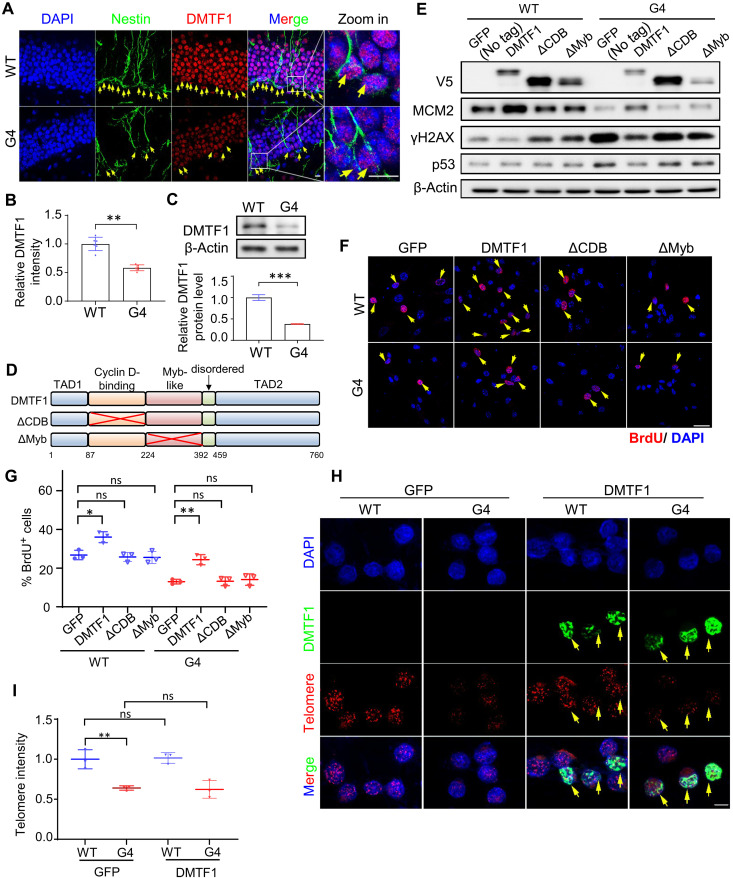
DMTF1 up-regulation rescues proliferation defect of telomere dysfunctional mouse NSCs. (**A**) Representative images of Nestin and DMTF1 immunofluorescence in WT and G4 TERT^ER/ER^ mouse NSCs in the dentate gyrus of hippocampus. Scale bar, 2 μm. Yellow arrows indicate Nestin^+^ cells. DAPI, 4′,6-diamidino-2-phenylindole. (**B**) Quantification of DMTF1 intensity in WT and G4 TERT^ER/ER^ mouse NSCs (*n* = 6 mice, 15 Nestin^+^ cells per mouse) (means ± SD). **P < 0.01. (**C**) Western blot analysis of DMTF1 levels in WT and G4 TERT^ER/ER^ mouse NSCs. β-Actin serves as the loading control. Quantification of DMTF1 band intensity when normalized to loading control is shown below (*n* = 3 replicates) (means ± SD). ***P < 0.001. (**D**) Structures of human DMTF1 protein and the variants that lack CDB (∆CDB) and Myb-like (∆Myb) domains. (**E**) Western blot analysis of V5, MCM2, γH2AX, and p53 in WT and G4 TERT^ER/ER^ mouse NSCs, with or without overexpression of V5-tagged WT, ΔCDB, or ΔMyb DMTF1. β-Actin serves as the loading control. (**F** and **G**) Representative images (F) and quantification (G) of BrdU^+^ cells in WT and G4 TERT^ER/ER^ mouse NSCs, with or without overexpression of WT, ΔCDB, or ΔMyb DMTF1. Scale bar, 20 μm. (*n* = 3 replicates, six fields/images per replicate) (means ± SD). ns, not significant, *P < 0.05, **P < 0.01. Yellow arrows indicate BrdU^+^ cells. (**H**) Representative images of V5 immunofluorescence and telomere probe fluorescence in WT and G4 TERT^ER/ER^ mouse NSCs, with or without DMTF1 overexpression. Scale bar, 5 μm. Yellow arrows indicate V5-DMTF1 overexpression cells. (**I**) Quantification of telomere intensity of WT and G4 TERT^ER/ER^ mouse NSCs, with or without DMTF1 overexpression. (*n* = 3 replicates, 10 cells per replicate) (means ± SD). ns, not significant, **P < 0.01.

Given the role of DMTF1 in regulating cell cycle progression (*16*) and its relatively high expression in the brain (*18*), we first explored whether DMTF1 expression may be dysregulated in the NSCs of telomerase-deficient mice (TERT^ER/ER^). The late generation (e.g., generation 4 or G4) telomerase-deficient mice have critically short telomeres that activate p53, leading to a marked reduction in NSC proliferation and neurogenesis (*5*). DMTF1 expression was substantially down-regulated in the NSC of G4 TERT^ER/ER^ mice in vivo and in vitro (Fig. 1, A to C). This finding was also recapitulated in TERT-deficient human neural progenitor cells (hNPCs) (fig. S1A) (*35*). Since telomere dysfunction induces DNA damage that activates p53 (*36*), we hypothesize that DMTF1 down-regulation may be indirectly attributed to p53 induction since DMTF1 is not a known p53 target gene (*37*). p53 depletion rescued DMTF1 protein expression in TERT-deficient hNPC (fig. S1A). We also show that DMTF1 down-regulation was not specific to telomeric damage as treatment with etoposide similarly reduced DMTF1 protein levels, in a p53-dependent manner (fig. S1B).

Next, we investigated the effect of DMTF1 overexpression on the proliferation of telomere dysfunctional NSCs. Although the CDB domain of DMTF1 binds to D-type cyclins that allows for CDK4-mediated DMTF1 phosphorylation, both CDB and Myb domains are crucial for DMTF1 binding to DNA (*38*, *39*). We found that the overexpression of WT DMTF1, but not the ΔCDB or ΔMyb mutants, fully rescued the proliferation defect of telomere dysfunctional mouse NSC as revealed by the frequency of 5-bromo-2′-deoxyuridine (BrdU^+^) cells and MCM2 protein levels (Fig. 1, D to G). This was associated with a decrease in γH2AX and p53 levels, consistent with the enhanced proliferation phenotype (Fig. 1E). DMTF1 overexpression also increased the proliferation of WT NSC (Fig. 1G). DMTF1 overexpression did not rescue telomeric defect in the telomere dysfunctional mouse NSC, excluding the possibility that the enhanced NSC proliferation was due to the restoration of eroded telomeres (Fig. 1, H and I). That DMTF1 overexpression can rescue telomere dysfunctional NSC proliferation was further confirmed using TERT-deficient hNPC, which also showed a decrease in γH2AX and p53 levels (fig. S1, C to E). Collectively, we demonstrate that DMTF1 down-regulation contributes to the NSC proliferation defect in the telomere dysfunctional mouse model. This finding was unexpected since DMTF1 is required for hematopoietic stem cell quiescence (*19*).

### DMTF1 loss drastically impairs mouse and human NSC proliferation in vitro

To strengthen our view that DMTF1 expression is associated with proliferative NSC, we compared DMTF1 expression in embryonic mouse NSCs that were cultured in the presence of bone morphogenetic protein 4 (BMP4) (to mimic the deep quiescent state), BMP4 + fibroblast growth factor (FGF) (to mimic the shallow quiescent state), or human epidermal growth factor (EGF) + FGF (to mimic the activated state) (*40*). It was clear that DMTF1, MCM2, and PCNA expression levels were the highest in NSCs cultured with EGF + FGF, indicating that DMTF1 expression is associated with NSC proliferation (fig. S2A). This was further supported by independent two single-cell RNA sequencing (RNA-seq) analyses of postnatal mouse NSC (*41*, *42*) wherein DMTF1 expression was higher in activated than quiescent NSC, and there were more DMTF1-expressing cells in the activated than quiescent NSC clusters (fig. S2, B to G). Notably, we observed the down-regulation of DMTF1 expression in all cells from the SVZ (one of the NSC niches) of old (22 months old) versus young (2 months old) mice from the Kalamakis dataset, particularly in the proliferative aNSC2 cluster (fig. S2, H and I). Furthermore, DMTF1 expression was slightly reduced in the dentate gyrus hippocampal regions of old (mean age 23.3 ± 1.89 years) versus young (mean age 7.04 ± 0.62 years) rhesus macaques (fig. S2J). These correlations suggest that DMTF1 down-regulation may also be associated with brain aging in naturally aged animals.

**Fig. 2.**
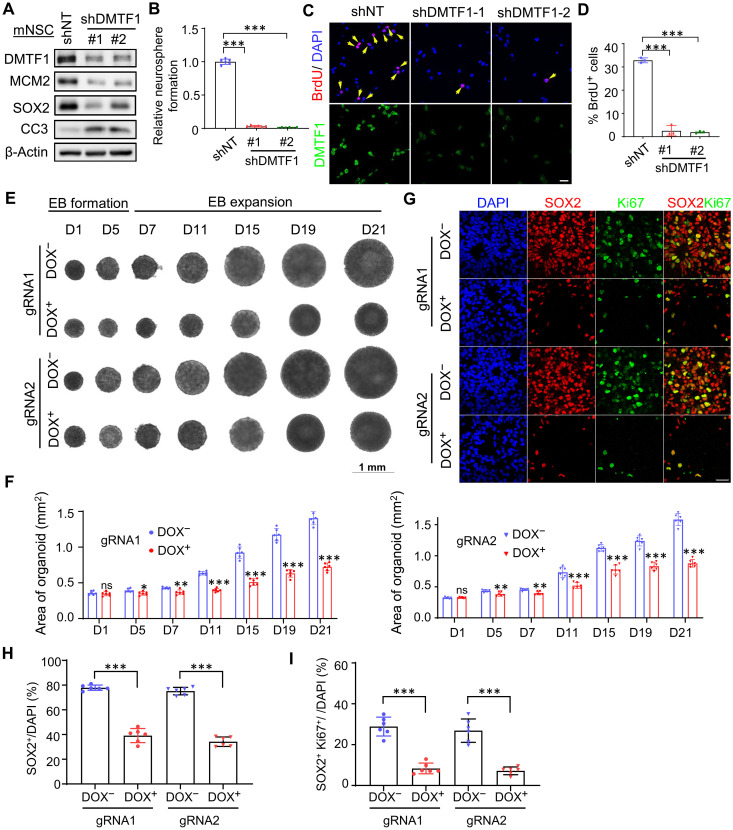
DMTF1 loss drastically impairs mouse and human NSC proliferation in vitro. (**A**) Western blot analysis of DMTF1, MCM2, SOX2, and cleaved-caspase 3 (CC3) levels in DMTF1 KD mouse NSCs. β-Actin serves as the loading control. (**B**) Neurosphere formation assay of DMTF1 KD mouse NSCs (*n* = 6 replicates) (means ± SD). ***P < 0.001. (**C**) Representative images of DMTF1 and BrdU immunofluorescence of DMTF1 KD mouse NSCs. Scale bar, 20 μm. Yellow arrows indicate BrdU^+^ cells. (**D**) Quantification of BrdU^+^ mouse NSCs upon DMTF1 KD (*n* = 3 replicates, 6 fields/images per replicate) (means ± SD). ***P < 0.001. (**E**) Brightfield images of cortical organoids at the indicated time points during the course of differentiation. The DMTF1 KO was induced by adding doxycycline (DOX). (**F**) Quantification of cortical organoid size in DMTF1 intact and KO cortical organoids (*n* = 6 organoids) (means ± SD). ns, not significant, *P < 0.05, **P < 0.01, ***P < 0.001. (**G**) Representative images of SOX2 and Ki67 immunofluorescence in DMTF1 intact and KO cortical organoids. Scale bar, 20 μm. (**H** and **I**) Quantification of SOX2^+^ (H) and SOX2^+^ Ki67^+^ (I) cells in cortical organoids upon DMTF1 KO. (*n* = 6 organoids) (means ± SD). ***P < 0.001.

Next, we used embryonic mouse and human NSCs, as well as human cortical organoids to determine the role of DMTF1 in NSC proliferation. DMTF1 depletion drastically reduced the protein levels of MCM2 (a proliferation marker) and SOX2 (an established NSC marker), neurosphere formation and BrdU^+^ cells, indicating decreased mouse NSC proliferation and stemness (Fig. 2, A to D). Consistent with a proliferation defect, DMTF1 knock down (KD) NSC exhibited a partial arrest in cell cycle progression with increased subpopulations in G_1_ phase and reduced S-G_2_ phases (fig. S3A). This was accompanied by an increase in apoptosis marked by elevated cleaved-caspase 3 protein levels in DMTF1 KD mouse NSC (Fig. 2A). DMTF1 silencing also reduced MCM2 protein level and BrdU^+^ cells, while increasing apoptosis of hNPCs (fig. S3, B to D). In human cortical organoids, DMTF1 loss [doxycycline treatment induced expression of single guide RNAs (sgRNAs) against DMTF1] substantially decreased the size of the organoids, which tracked closely with the number of NSC (SOX2^+^), proliferating NSC (SOX2^+^Ki67^+^), and neuroepithelial structures (ZO-1^+^) (Fig. 2, E to I, and fig. S3, E to G). Similar to the mouse NSC and hNPC, we observed a decrease in MCM2 but an increase in cleaved-caspase 3 protein levels in DMTF1 KO organoids (fig. S3E). To exclude the possibility that doxycycline treatment alone affected human ESC (hESC) growth, we treated hESC with doxycycline (1 μg/ml) for 3 days and then determined cell confluency as well as DMTF1 and MCM2 protein levels. There was no change in cell confluency, as well as protein levels of DMTF1 and MCM2 in doxycycline-treated hESC (fig. S3, H and I), indicating that doxycycline treatment alone did not reduce hESC proliferation and cannot contribute to the proliferation defect of DMTF1 KO cortical organoids. Contrary to its proposed role as a tumor suppressor in cancer, we provide compelling evidence that DMTF1 acts to promote mouse and human NSC proliferation in vitro.

### DMTF1 serves as a transcriptional activator of chromatin organization genes in mouse NSC

**Fig. 3.**
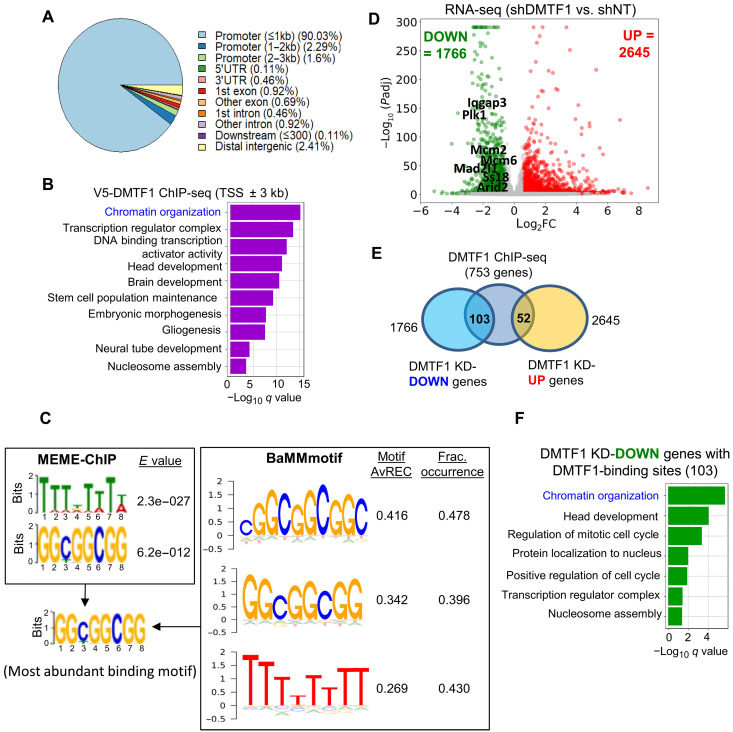
DMTF1 serves as a transcriptional activator of chromatin organization genes in mouse NSC. (**A**) Genomic distribution of V5-DMTF1 ChIP-seq peaks in mouse NSCs. Total number of detected DMTF1 peaks: 873. (**B**) Pathway enrichment analysis of genes proximal to the V5-DMTF1 ChIP-seq peaks at gene promoters (±3 kb from gene TSS). (**C**) De novo motif analysis of V5-DMTF1 peaks using MEME-ChIP and BaMMmotif software. (**D**) RNA-seq analysis of DMTF1 KD mouse NSCs showing the number of differentially expressed genes. (**E**) Intersection of genes associated with DMTF1 ChIP-seq peaks (753 genes) with differentially expressed genes in DMTF1 KD mouse NSCs. (**F**) Gene Ontology enrichment analysis of the 103 genes in (E).

To determine the genome-wide binding profile of DMTF1 in NSC, we stably expressed a V5-tagged DMTF1 for subsequent V5 antibody–mediated chromatin immunoprecipitation sequencing (ChIP-seq) as there is no commercially available ChIP grade DMTF1 antibody. We first validated the functionality of the exogenously expressed V5-DMTF1 by performing a genetic complementation experiment involving the overexpression of WT, ΔCDB, or ΔMyb V5-DMTF1 in DMTF1-depleted mouse NSC. As expected, the overexpression of WT DMTF1, but not the ΔCDB and ΔMyb mutants, rescued the proliferation defect of DMTF1-silenced mouse NSC based on MCM2 levels and frequency of BrdU^+^ cells (fig. S4, A to C). Following this, V5 antibody–mediated ChIP-seq analysis of V5-tagged DMTF1-expressing NSC (without DMTF1 KD) revealed that the vast majority of DMTF1 ChIP-seq peaks were enriched at the promoter regions (within ±1 to 3 kb from the transcription start site) (>93%) (Fig. 3A). Pathway enrichment analysis of the DMTF1 proximal genes showed the significant enrichment of pathways, including “chromatin organization” (Fig. 3B). To identify de novo motifs in our DMTF1 ChIP-seq data, we next used the MEME-ChIP and BaMMmotif software. Both software predicted the significant enrichment of “GGCGGCGG” motifs in about 48% of the DMTF1-binding sites (Fig. 3C). In contrast, we do not find significant enrichment of the reported DMTF1 motif “CCCGCATGC” (*38*) in our DMTF1 ChIP-seq dataset. Next, we sought to identify high-confidence DMTF1 target genes that can account for DMTF1’s role in promoting mouse NSC proliferation. RNA-seq analysis identified 1766 down-regulated and 2645 up-regulated genes in DMTF1-depleted mouse NSC as compared to control NSC (Fig. 3D). Integrative analysis of the RNA-seq and DMTF1 ChIP-seq datasets then revealed 103 down-regulated and 52 up-regulated bona fide DMTF1 target genes (Fig. 3E). Notably, pathway enrichment analysis of the 103 down-regulated DMTF1 target genes showed the significant enrichment of chromatin organization as the top pathway (Fig. 3F). We conclude that DMTF1 serves as a transcriptional activator of chromatin organization genes in mouse NSC.

### Identification of Arid2 and Ss18 as bona fide DMTF1 gene targets in mouse NSC

**Fig. 4.**
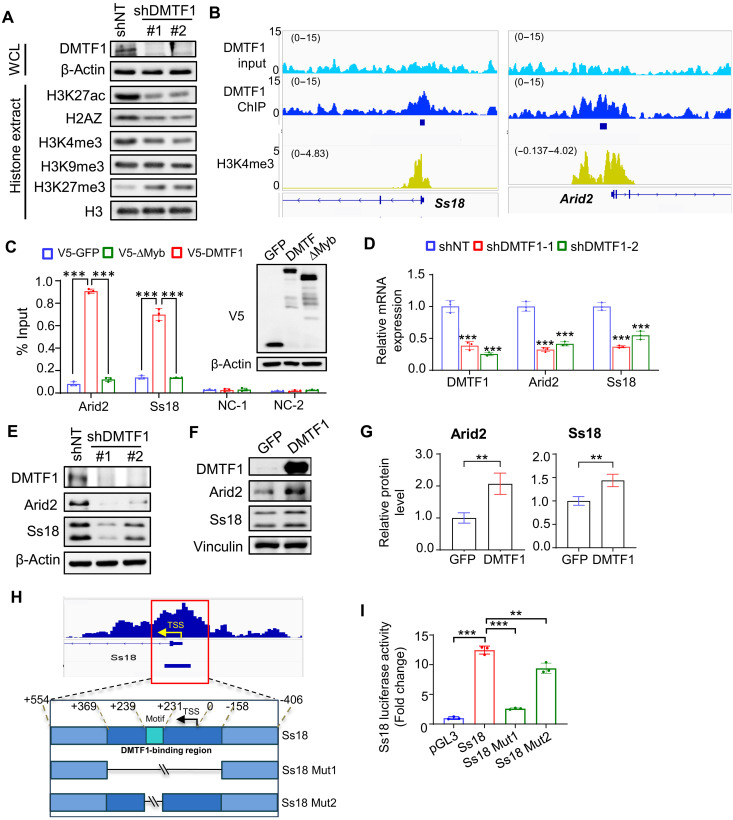
Identification of Arid2 and Ss18 as bona fide DMTF1 gene targets in mouse NSC. (**A**) Western blot analysis of protein levels of DMTF1 in whole-cell lysate (WCL) and selected histone modifications in histone extracts of DMTF1 KD mouse NSCs. β-Actin serves as the loading control for WCL, while H3 serves as the loading control for histone extracts. (**B**) DMTF1 and H3K4me3 ChIP-seq tracks of *Ss18* and *Arid2*. (**C**) ChIP-qPCR analysis of DMTF1 occupancy at the promoter of *Arid2* and *Ss18* in mouse NSCs (*n* = 3) (means ± SD). NC, negative control primer, ***P < 0.001. Western blot analysis of V5-tagged GFP, DMTF1, and ΔMyb levels upon overexpression in mouse NSCs, which were used in the ChIP-qPCR experiment (shown on the right). β-Actin serves as the loading control. (**D**) qPCR analysis of Arid2 and Ss18 mRNA levels in DMTF1 KD mouse NSCs. (*n* = 3 replicates) (means ± SD), ***P < 0.001. (**E**) Western blot analysis of DMTF1, Arid2, and Ss18 protein levels in DMTF1 KD mouse NSCs. β-Actin serves as the loading control. (**F**) Western blot analysis of DMTF1, Arid2, and Ss18 protein levels in DMTF1-overexpressing mouse NSCs. Vinculin serves as the loading control. (**G**) Quantification of Arid2 and Ss18 band intensity when normalized to loading control in (F) (*n* = 3 replicates) (means ± SD). **P < 0.01. (**H**) Schematic diagram showing the WT *Ss18*, *Ss18* Mut1 and *Ss18* Mut2 genomic regions used in the luciferase reporter assay. (**I**) Ss18 luciferase activity when constructs containing the *Ss18* genomic regions in (H) were used to drive luciferase expression in HEK293T cells. (*n* = 3 replicates) (means ± SD). **P < 0.01, ***P < 0.001.

To prioritize which chromatin organization genes may be important for DMTF1’s function, we conducted Western blotting of a subset of histone modifications using the histone extracts from DMTF1-depleted mouse NSC. DMTF1 silencing led to reduced levels of active H3K27ac, H2AZ, and H3K4me3 histone modifications but increased repressive H3K27me3 mark (Fig. 4A). As the SWI/SNF complex has been shown to maintain lineage-specific enhancers (*25*), reduced H3K27ac suggests that DMTF1 might regulate the expression level of some of its components. This hypothesis is consistent with the slight increase in Polycomb-associated H3K27me3 that occurs upon the loss of SWI/SNF subunits (*23*, *24*). Arid2 and Ss18 that are subunits of the SWI/SNF complexes were among the 103 DMTF1 target genes in the chromatin organization pathway (fig. S5A). In the V5 antibody–mediated ChIP-seq analysis, DMTF1 was visibly enriched at the promoter of *Arid2* and *Ss18* (Fig. 4B). To rule out nonspecific binding of V5-tag, promoter enrichment of V5-DMTF1 at *Arid2* and *Ss18* genes were compared against the V5–green fluorescent protein (GFP) control or V5-ΔMyb mutant, which were expressed at similar levels (Fig. 4C). ChIP–quantitative polymerase chain reaction (qPCR) showed that only V5-DMTF1 was enriched at the promoter of *Arid2* and *Ss18* genes in mouse NSC (Fig. 4C). We validated the reduction in mRNA and protein levels of Arid2 and Ss18 in DMTF1 KD NSC (Fig. 4, D and E). Consistent with our previous observation that DMTF1 was down-regulated in the G4 TERT^ER/ER^ NSC, Arid2 and Ss18 protein levels were also notably lower in the G4 TERT^ER/ER^ than WT NSC (fig. S5, B and C). Conversely, DMTF1 overexpression increased Arid2 and Ss18 protein levels in NSC (Fig. 4, F and G).

To validate our binding enrichment analysis, we chose to focus on Ss18 and asked whether the identified DMTF1-enriched sequence and GGCGGCGG motif can regulate Ss18 gene transcription. Using luciferase reporter assay, we found that the WT *Ss18* sequence increased luciferase activity by ~12-fold when compared to the empty pGL3 vector, but this effect was dramatically abolished with the *Ss18* Mut1 sequence (Fig. 4, H and I). However, the *Ss18* Mut2 sequence was still able to increase luciferase activity by ~ninefold, which was notably lower than the WT *Ss18* sequence (~25% lower than WT), suggesting that the GGCGGCGG motif contributes in part to gene transcription of Ss18 (Fig. 4I). It is important to point out that the GGCGGCGG motif can also be found in the Arid2 gene promoter within our DMTF1 ChIP-seq peak (fig. S5D). Together with our previous binding and expression data, these findings strengthen our view that Ss18 and Arid2 are bona fide DMTF1 gene targets in mouse NSC.

We also tested whether reduced Arid2 and Ss18 might be caused by defective cell cycle progression in DMTF1 KD NSC via two methods. First, transient inhibition of E2F1 by CDK4/6 inhibitor palbociclib did not substantially alter the levels of Arid2 and Ss18 mRNAs (NB: Mcm2 serves as a positive control for E2F1 gene) or H3K27ac mark (fig. S5, E and F). Second, silencing DMTF1 in slow-cycling NSC (cultured with BMP4 + FGF) decreased the mRNA levels of Arid2 and Ss18, but not Mcm2 (fig. S5G). In contrast, DMTF1 depletion in proliferating NSC (cultured with FGF + EGF) reduced the levels of Arid2, Ss18, and Mcm2 mRNAs (fig. S5G). These results suggest that the reduced levels of Arid2 and Ss18 were unlikely caused by proliferative defect of DMTF1 KD NSC.

### Attenuated E2F program is likely mediated by Arid2 and Ss18 in DMTF1 KD mouse NSC

**Fig. 5.**
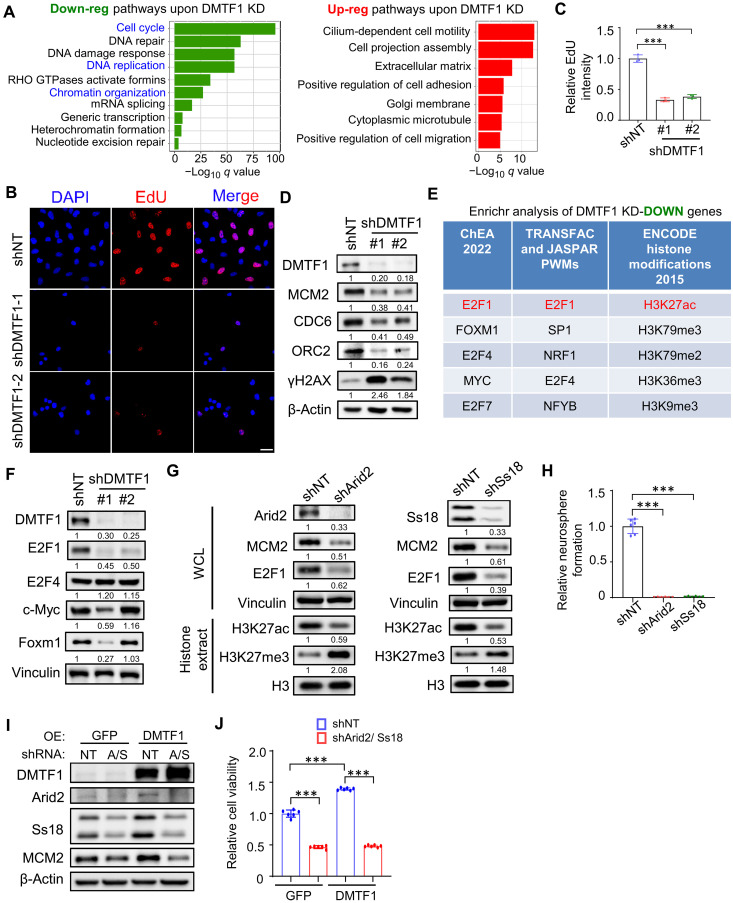
Attenuated E2F1 program is likely mediated by Arid2 and Ss18 in DMTF1 KD mouse NSC. (**A**) Pathway enrichment analysis of the down- and up-regulated genes in DMTF1 KD mouse NSCs. (**B** and **C**) Representative images (B) and quantification (C) of EdU immunofluorescence in DMTF1 KD mouse NSCs. Scale bar, 20 μm. (*n* = 3 replicates, 30 cells per replicate) (means ± SD). ***P < 0.001. (**D**) Western blot analysis of DMTF1, MCM2, CDC6, ORC2, and γH2AX levels in DMTF1 KD mouse NSCs. β-Actin serves as the loading control. The numbers indicate the relative band intensity of a protein when normalized to the shNT. (**E**) Enrichr analysis of down-regulated genes in DMTF1 KD mouse NSCs. (**F**) Western blot analysis of E2F1, E2F4, c-Myc, and Foxm1 levels in DMTF1 KD mouse NSCs. Vinculin serves as the loading control. The numbers indicate the relative band intensity of a protein when normalized to the shNT. (**G**) Western blot analysis of Arid2, Ss18, MCM2, and E2F1 levels in WCL, as well as H3K27ac and H3K27me3 levels in histone extracts of Arid2/ Ss18 KD mouse NSCs. Vinculin serves as the loading control for WCL, while H3 serves as the loading control for histone extracts. The numbers indicate the relative band intensity of a protein when normalized to the shNT. (**H**) Neurosphere formation assay of Arid2 or Ss18 KD mouse NSCs (*n* = 6 replicates) (means ± SD). ***P < 0.001. (**I**) Western blot analysis of DMTF1, Arid2, Ss18, and MCM2 levels in DMTF1-overexpressing WT mouse NSCs, with or without Arid2 and Ss18 depletion. β-Actin serves as the loading control. (**J**) Cell viability of DMTF1-overexpressing WT mouse NSCs, with or without Arid2 and Ss18 depletion (*n* = 6 replicates) (means ± SD). ***P < 0.001.

Pathway enrichment analysis was next performed to better understand transcriptomic changes caused by DMTF1 depletion. The down-regulated genes were enriched for “cell cycle,” “DNA replication” and “chromatin organization” pathways, whereas the up-regulated genes were associated with “cilium-dependent cell motility” and “cell projection assembly” pathways (Fig. 5A). In line with the altered transcriptional programs, we previously showed that DMTF1 KD NSC exhibited a partial arrest in cell cycle progression (fig. S3A). Down-regulation of DNA replication genes was also reflected by a significant reduction in 5-ethynyl-2′-deoxyuridine (EdU) labeling, as well as lower levels of prereplication complex proteins (e.g., MCM2, CDC6, and ORC2) and increased γH2AX levels in DMTF1 KD mouse NSC (Fig. 5, B to D). Notably, Enrichr analysis revealed E2F1, Foxm1, E2F4, and c-Myc as the top transcription factors with known roles in cellular proliferation, and H3K27ac as the top histone modifications for the down-regulated genes (Fig. 5E). Among the four candidate transcription factors, we found that only E2F1 levels reproducibly decreased in DMTF1 KD NSC (Fig. 5F). The silencing of either Arid2 or Ss18 phenocopied the reduction of MCM2/E2F1/H3K27ac levels and neurosphere formation as well as elevated H3K27me3 levels as observed in DMTF1 depleted mouse NSC (Fig. 5, G and H). By focusing on a subset of E2F target genes that were down-regulated in DMTF1-depleted NSC (from our RNA-seq), we also showed that the mRNA levels of Plk1, Mad2l1, lqgap3, Mcm6, and Mcm2 were notably reduced upon Arid2, Ss18, or DMTF1 silencing in NSC (fig. S6, A to C). To test the functional link between DMTF1 and Arid2/Ss18, we depleted Arid2 and Ss18 in DMTF1 overexpressing WT and G4 TERT^ER/ER^ NSC, and assessed NSC proliferation based on MCM2 protein level and cell viability. The increased proliferation of DMTF1 overexpressing WT and G4 TERT^ER/ER^ NSC was abrogated upon silencing of Arid2/Ss18 (Fig. 5, I and J, and fig. S6, D and E). These findings indicate that the attenuated E2F program is likely mediated by Arid2 and Ss18 in DMTF1 KD mouse NSC.

### Reduced Arid2 and Ss18 binding at E2F targeted promoters for gene activation in DMTF1-deficient NSC

**Fig. 6.**
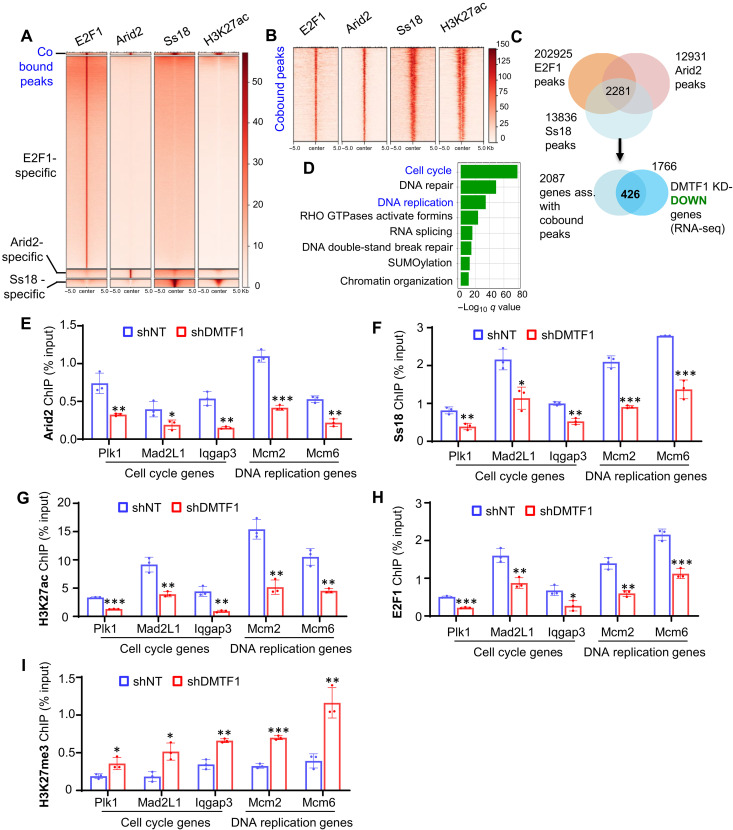
Absence of DMTF1 reduces Arid2 and Ss18 occupancy at the promoter of E2F genes, leading to concomitant loss of gene activation and expression in mouse NSC. (**A** and **B**) Heatmaps indicating the cobound, E2F1-, Arid2-, Ss18-specific clusters and their H3K27ac-binding profiles. (**C**) Venn diagram showing the number of down-regulated genes in DMTF1 KD mouse NSC with Arid2/Ss18/E2F1 co-occupancy. (**D**) Pathway enrichment analysis of the 426 genes in (C). (**E** to **I**) ChIP-qPCR analysis of Arid2 (E), Ss18 (F), H3K27ac (G), E2F1 (H), and H3K27me3 (I) enrichment at the promoter of representative cell cycle (*Plk1*, *Mad2L1*, and *Iqgap3*) and DNA replication (*Mcm2* and *Mcm6*) genes in DMTF1 KD mouse NSCs. (*n* = 3 replicates) (means ± SD), *P < 0.05, **P < 0.01, ***P < 0.001.

Our results suggest a model whereby DMTF1 activates the transcription of Arid2 and Ss18, two subunits of SWI/SNF complexes, which in turn modulate H3K27ac levels at the promoters of E2F target genes to facilitate gene activation (fig. S7A). Such a model would predict the colocalization of either Arid2 or Ss18 with E2F and H3K27ac mark at the promoters of cell cycle and DNA replication genes. To test this, publicly available ChIP-seq datasets of Arid2, Ss18, H3K27ac, E2F1, and E2F4 were downloaded for unbiased genome-wide analysis. In agreement with Arid2 and Ss18 belonging to different SWI/SNF complexes (*21*), most Arid2- and Ss18-bound regions are mutually exclusive (Fig. 6A). However, we found a subset of Arid2- and Ss18-bound regions (i.e., “shared”) that appeared to be enriched on the H3K27ac marks (Fig. 6, A and B). These shared regions also contained E2F1 and E2F4 peaks, which mapped to about 2087 Arid2/Ss18/E2F1 cobound genes and 732 Arid2/Ss18/E2F4 cobound genes (Fig. 6C and fig. S7B). DMTF1 depletion led to the down-regulation of 426 of the 2087 E2F1/Arid2/Ss18 cobound genes and 286 of the 732 Arid2/Ss18/E2F4 cobound genes (Fig. 6C and fig. S7B). In agreement with E2F1 and E2F4 having key roles in promoting transcription of cell cycle genes (*43*), the 426 E2F1/Arid2/Ss18 cobound genes and 286 Arid2/Ss18/E2F4 cobound genes were enriched in cell cycle and DNA replication pathways (Fig. 6D and fig. S7C). We chose to focus on E2F1 for our subsequent epigenomic analyses as (i) E2F1 but not E2F4 levels decreased in DMTF1 KD NSC (Fig. 5F), and (ii) there were more E2F1/Arid2/Ss18 than E2F4/Arid2/Ss18 gene targets that were down-regulated in DMTF1 KD NSC (24.1 versus 16.2%) (Fig. 6C and fig. S7B). A subset of cell cycle (e.g., Plk1, Mad2L1, and lqgap3) and DNA replication (e.g., Mcm6 and Mcm2) genes were blown-up to show the detailed co-occupancy patterns of Arid2, Ss18, E2F1, active H3K27ac, and H3K4me3 marks in fig. S7 (D and E).

We next analyzed the binding patterns of Arid2, Ss18, E2F1, H3K27ac, and H3K27me3 at these cell cycle and DNA replication genes using ChIP-qPCR in control and DMTF1 KD NSC. There were substantially lower levels of Arid2, Ss18, H3K27ac, and E2F1 enrichment at the promoter of Plk1, Mad2l1, lqgap3, Mcm6, and Mcm2 in DMTF1 KD NSC (Fig. 6, E to H). Consistent with reduced SWI/SNF activity at the promoter of these genes, there was a significant increase in H3K27me3 levels at the promoter of Plk1, Mad2l1, lqgap3, Mcm6, and Mcm2 in DMTF1 silenced NSC, likely due to increased Polycomb complex activity as reported previously (Fig. 6I) (*23*). Thus, the absence of DMTF1 reduces Arid2 and Ss18 occupancy at the promoter of E2F genes, leading to concomitant loss of gene activation and expression in mouse NSC.

## DISCUSSION

In this study, we serendipitously unveil that DMTF1 is down-regulated in NSCs that experience genotoxic stress, including dysfunctional telomeres. Intriguingly, DMTF1 up-regulation is sufficient to rescue the proliferation defect of telomere dysfunctional NSC. This finding is exciting as the molecular underpinnings of how telomere dysfunction compromises NSC proliferation remain largely unknown and there are only a small handful of examples that can overcome telomere dysfunction-induced cell cycle arrest, including p53 and Batf3 (for hematopoietic stem cells) (*36*, *44*). In contrast to previous studies showing that DMTF1 LOH or null promotes tumorigenesis in Eμ-myc–induced B cell lymphoma, *K-ras^LA^* lung cancer and MMTV-neu/Erbb2 breast cancer models (*13*–*15*), we now report a growth promoting function of DMTF1 in NSC. This aligns with the observation that targeted disruption of DMTF1 in mice results in very small mice at birth and the death of about one-third of such DMTF1 null mice (*18*). Only one of 40 DMTF1 null mice spontaneously developed a tumor in the first year of life, indicating that DMTF1 deficiency alone is insufficient to initiate tumorigenesis (*18*). These observations suggest that DMTF1 may play a tumor suppressive or promoting role in a cell type–/context-dependent manner.

**Fig. 7.**
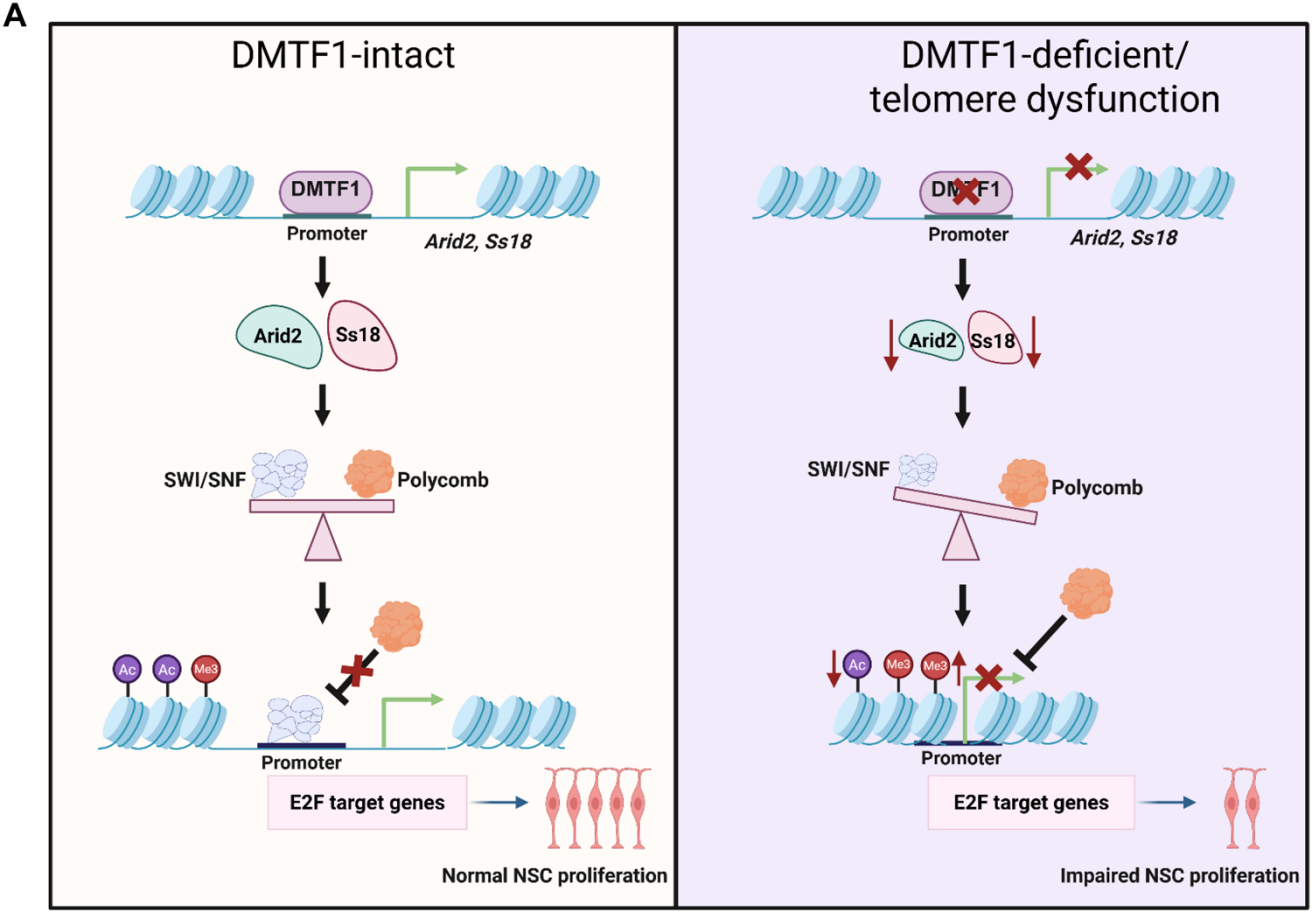
Proposed model of how DMTF1 promotes NSC proliferation by regulating the SWI/SNF-E2F axis. In WT NSC, DMTF1 promotes gene transcription of Arid2 and Ss18, which then bind to the promoter of E2F gene targets where they modulate H3K27ac levels, for gene activation. In DMTF1-deficient or telomere dysfunctional NSC, Arid2 and Ss18 deficiency reduces H3K27ac levels while increases Polycomb-associated H3K27me3 levels at E2F targets, leading to the repression of cell cycle and DNA replication genes.

Our mechanistic investigations reveal that DMTF1 promotes NSC proliferation in part by regulating the transcription of Arid2 and Ss18, subunits of the PBAF and cBAF complexes, respectively, which control chromatin accessibility of specific transcription factors. This is backed by the enrichment of DMTF1 (but not GFP control or ΔMyb mutant) at the promoter of *Arid2* and *Ss18*, down-regulation of Arid2 and Ss18 at the mRNA and protein levels in DMTF1 KD NSC, and perturbed balance of H3K27ac and H3K27me3 marks upon DMTF1 silencing (which is reminiscent of SWI/SNF subunit loss). Given the modest overlap between transcription factor binding and gene regulation as shown by systematic analysis of the near-complete set of transcription factors in yeast (*45*), the mutational analysis of enhancers and promoters is essential for proper interpretation of bona fide gene targets. By using the luciferase reporter assay, we demonstrated that our identified DMTF1 binding region and putative DMTF1 motif (GGCGGCGG) contributes to the gene transcription of Ss18, supporting our idea that Ss18 (and likely Arid2) is a bona fide DMTF1 target gene. Our findings add to the small handful of reported DMTF1 gene targets [including Arf and CD13/aminopeptidase N (*16*, *46*)] and represent a genome-wide assessment of DMTF1’s DNA binding profile in mouse NSC. We propose that Arid2 and Ss18 bind to the promoter of E2F gene targets where they modulate H3K27ac levels, for gene activation in DMTF1-intact NSC (Fig. 7). In DMTF1-deficient (or telomere dysfunctional) NSC, Arid2 and Ss18 deficiency reduces H3K27ac levels while increases Polycomb-associated H3K27me3 levels at E2F targets, leading to the repression of cell cycle and DNA replication genes. Consistent with this model, Arid2 or Ss18 KD phenocopied DMTF1 loss in decreasing H3K27ac and E2F1 levels, as well as expression of cell cycle and DNA replication genes, while augmenting H3K27me3 levels.

Although Ss18 and Arid2 belong to different SWI/SNF complexes, we found shared Ss18- and Arid2-bound genomic regions, which colocalize with the H3K27ac marks (Fig. 6, A and B). This observation corroborates with a previous study showing that PBAF and BAF have distinct yet overlapping genomic occupancy in melanoma (*47*). Specifically, the PBAF-BAF shared regions (Arid2- and Ss18-bound) are enriched at the promoter of active genes and display BRG1 binding. Furthermore, PBAF plays a key role in maintaining open chromatin at a subset of Arid2-bound sites in both PBAF and shared regions. Consistent with the idea that Arid2 loss can disrupt SWI/SNF activity, Arid2 loss leads to deficient PBAF complex assembly in melanoma cells. While Arid2 loss can result in genomic redistribution of BAF in melanoma, this is unlikely to occur in DMTF1 KD NSC given that DMTF1 loss down-regulates both Ss18 and Arid2 expression.

Cell cycle progression is crucial for cellular proliferation, and its dysregulation can lead to carcinogenesis. Hence, the mammalian cell cycle must be carefully coordinated, and this involves a variety of mechanisms, including the precise activation of CDKs in response to growth stimuli, degradation of specific cell cycle proteins through the ubiquitin-proteasome system, and the transcriptional regulation of cell cycle genes by E2F, B-MYB, and FOXM1 transcription factors (*48*). Pertinent to this study, E2F1 and E2F4 play key roles in the G_1_-S cell cycle progression. Cyclin D:CDK1/2 can hyperphosphorylate retinoblastoma (RB), which causes RB dissociation from activator E2Fs and exposes the transactivation domain of E2Fs. The activating E2Fs can recruit chromatin modifiers, including Tip60 and PCAF/GCN5, to activate G_1_-S genes that are in the DNA metabolism, DNA replication, and DNA repair pathways (*49*). The SWI/SNF subunits, Arid1a and Arid1b, can interact with different E2F proteins to differentially influence cell cycle progression. While Arid1b associates with E2F1 (along with a histone acetyltransferase) to activate cell cycle gene expression, Arid1a binds to repressive E2Fs (along with a histone deacetylase) to repress cell cycle genes. Thus, our study has unraveled a previously unidentified level of E2F regulation that involves DMTF1-mediated transcriptional control of SWI/SNF genes that subsequently confers an E2F-permissive chromatin state.

DMTF1 can regulate the Arf-p53 pathway that is central to the cellular response to genotoxic stress, including telomere dysfunction. On the other hand, the alternative lengthening of telomeres (ALT) is often activated in cells with telomerase deficiency and p53 pathway alterations. Given that DMTF1 can transcriptionally regulate the SWI/SNF-E2F axis (which in turn influences cell cycle progression and genome stability) and that activating E2Fs can recruit PCAF/GCN5, which have opposite effects on ALT maintenance (*50*), DMTF1 loss may alter telomeric chromatin remodeling that is a fundamental mechanism in the ALT pathway. The loss of ATRX, a member of the SWI/SNF family that serves as a histone H3.3–specific chaperone, progressively induces telomere DNA replication dysfunction that activates homology-directed DNA repair responses and cell cycle checkpoint control (*51*). This helps to explain why ATRX loss is often observed in human cancers that use the ALT pathway for chromosomal end protection. Thus, it is tempting to speculate a potential convergence of DMTF1 in both telomerase and ALT mechanisms, which may have important implications in aging and cancer research.

In summary, we have demonstrated that DMTF1 up-regulation is sufficient to rescue the proliferation defect of telomere dysfunctional NSCs and unveiled a previously unrecognized role of DMTF1 in regulating SWI/SNF gene transcription for NSC proliferation. Our study has several limitations that may prevent its immediate translation. While we showed that DMTF1 overexpression can rescue the proliferation defect of telomere dysfunctional NSC in vitro, whether DMTF1 up-regulation enhances NSC functional pool (e.g., neurogenesis) in telomere dysfunctional mice or naturally aged mice remains unknown. Furthermore, it is unclear whether DMTF1 up-regulation may increase the tumorigenic potential of NSC. Although DMTF1 is notably up-regulated in glioblastoma (GBM) when compared to the nontumor brain tissues in three independent glioma datasets, DMTF1 expression failed to predict the outcome of patients with glioma (fig. S8, A and B), suggesting that DMTF1 may not have clinical relevance in GBM. These issues would have to be addressed before we consider strategies that boost DMTF1 activity as an NSC rejuvenation therapy for the aged brain.

## MATERIALS AND METHODS

### Mouse models

TERT^ER/ER^ (i.e., an estrogen receptor–TERT fusion protein that is inactive unless with 4-hydroxytamoxifen (4-OHT) treatment) mice were provided by R. DePinho (MD Anderson Cancer Center) under a materials transfer agreement. The intergenerational crossing of these telomerase deficient mice yields late generation (fourth generation or G4) mice with critically short telomeres that activate p53, leading to widespread tissue degeneration (NB: mice have long telomeres). Genotyping was performed by PCR as previously described (*5*). All samples were collected at postnatal day 60 (P60). The animal care and experimental procedures previously described in (*52*), which were approved by and performed under guidelines provided by the Institutional Animal Care and Use Committee of the National University of Singapore and complied with the Association for Assessment and Accreditation of Laboratory Animal Care guidelines for animal use (BR17-0016, R17-0058, BR20-1577, and R21-0362). The day of identifying a vaginal plug and the day of birth were designated as embryonic day 0.5 (E0.5), and P0, respectively. All mice used in this study were housed in groups in individually ventilated cages under a 12:12-hour light/dark cycle with access to food and water. Because there was no apparent gender bias in the observed phenotypes and pathology, female and male mice were included and randomly allocated to experimental groups according to age and genotype.

### Immunofluorescence

Following procedures previously described in (*52*), for tissue immunofluorescence, the brain tissue was fixed in 4% paraformaldehyde (PFA) for 15 min, cryopreserved in 30% sucrose for 48 hours, and embedded in Surgipath FSC 22 Clear Frozen Section Compound. Then, the mouse brain tissue (day 60) was sectioned at 25 μm floating in 1× phosphate-buffered saline (PBS), and the cortical organoid (day 21) was sectioned at 10 μm on slides using a cryostat. The tissue was permeabilized with 0.3% PBS-T for 15 min and then incubated for 30 min at 90°C in citrate antigen retrieval buffer at pH 6. After cooling down at room temperature (RT), the tissue was blocked in the blocking buffer consisting of 3% bovine serum albumin (BSA) in 1× PBS-T. After 1 hour of blocking, the sections were incubated with primary antibodies (table S1) at 4°C overnight. The tissue was washed with 0.3% PBS-T three times and then incubated in secondary antibody at RT for 2 hours in the dark. After washing with 0.3% PBS-T three times, the slide was mounted with Prolong Gold anti-fade (Thermo Fisher Scientific) and observed using confocal (Zeiss, LSM710) with appropriate filters.

For cell immunofluorescence, the cells were fixed in 4% PFA at RT for 15 min and washed in 1× PBS for 5 min. For BrdU staining, the cells were incubated with 10 μM BrdU (Roche) for 1 hour at 37°C before fixation.

### Cell lines and compounds

Human embryonic kidney (HEK) 293T cells were cultured in Dulbecco’s modified Eagle’s medium (DMEM)–high glucose (Nacalai tesque) with 10% fetal bovine serum (Thermo Fisher Scientific) and 1% penicillin-streptomycin (PS). HEK 293T cells were passaged using trypsin (Thermo Fisher Scientific). Human H1 ESCs were provided by M. Pouladi (UBC Medical Genetics Department). The ESCs were cultured in mTeSR1 media (STEMCELL), and the cells were passaged using ReLeSR (STEMCELL). Human H1 NPCs were induced from H1 ESCs using Neurobasal Plus Medium (Thermo Fisher Scientific) and DMEM/Ham’s F-12 medium (Nacalai tesque), which were supplemented with BSA (5 μg/ml), 1× GlutaMax, 1× PS, 1× N2, 1× B27 without vitamin A, human leukemia inhibitory factor (10 ng/ml), 4 μM CHIR99021, 3 μM SB431542, and 0.1 μM Compound E. The H1 NPCs were cultured in human ReNcell NSC Maintenance Media (Sigma-Aldrich), which was supplemented with human EGF (20 ng/ml), human basic FGF (human FGF, 20 ng/ml), and 1% PS. The H1 NPCs were passaged using Accutase (Sigma-Aldrich).

Mouse NSCs were isolated from the embryo cortex (E14.5) of WT and G4 TERT^ER/ER^ mice. The cortex was dissociated using the Neural Tissue Dissociation Kit (Miltenyi). The mouse NSCs were cultured in NeuroCult Proliferation mouse NSC media (STEMCELL), which was supplemented with human EGF (20 ng/ml), human FGF (20 ng/ml), and 1% PS. The mouse NSCs were passaged using Accutase (Sigma-Aldrich). Quiescence was induced by culturing the mouse NSC in NSC media containing BMP4 (10 ng/ml, Peprotech), without growth factors (*40*) for more than 3 days. The etoposide treatment for mouse NSCs is 5 μM for 3 hours. All cells were cultured in a 5% CO_2_ incubator at 37°C.

### Lentiviral transduction

Following procedures previously described in (*52*), the lentivirus was produced using a third-generation system. HEK 293T cells were cotransfected with pMD2.G, pRSV-Rev, pMDVSVG, and overexpression/short hairpin RNA (shRNA) plasmids. After 72 hours, the lentivirus was harvested and concentrated by ultracentrifuge (Optima, XL-100 K). The lentiviral particles were then resuspended using DMEM/F12 (Nacalai tesque). The hESCs, hNPCs, and mouse NSCs were infected with lentivirus when splitting, and fresh media were replaced 24 hours after infection.

### Neurosphere formation assay

The neurosphere formation assay involved seeding mouse NSCs at a density of 5 cells per μl, and the number of neurosphere in each well was quantified after 5 days. The data presented are from 12 replicates.

### In vitro EdU labeling

Following procedures previously described in (*52*), the mouse NSCs were seeded onto coverslips and incubated with 10 μM EdU (Toronto Research Chemicals) for 1 hour at 37°C. The cells were fixed in 4% PFA at RT for 15 mins and washed in 1× PBS for 5 min. The slide was then permeabilized with 0.3% Triton X-100 in 1× PBS (PBS-T) for 15 min. The cells were then stained with EdU staining solution [100 mM tris (pH 7.5), 4 mM CuSO_4_, sulfo-cyanide azide (1 mg/ml), and 100 mM sodium ascorbate] for 1 hour at RT. After washing with 0.3% PBS-T three times, the slide was mounted with Prolong Gold anti-fade (Thermo Fisher Scientific) and observed using confocal (Zeiss, LSM710) with appropriate filters.

### CRISPR-Cas9 genome editing

The CRISPR-Cas9 genome editing system used is a two-vector system. The gRNA of DMTF1 was designed using online resources from Zhang Feng laboratory (https://addgene.org/pooled-library/zhang-human-gecko-v2/). The LentiV_Cas9_puro (Addgene, #108100) virus was transduced into hESC, followed by puromycin selection, after which single clones of hESC with Cas9 were used for the expression of sgRNA. The sgRNAs were cloned into FgH1tUTG plasmid (Addgene, #70183), which allows for the inducible expression of sgRNA with GFP reporter. The virus was transduced into hESCs that show high Cas9 expression. After sorting with GFP, doxycycline (1 μg/ml) was added to the cells for 3 days induce the expression of DMTF1 sgRNA. The sgRNA sequences used for CRISPR-Cas9 are listed in table S2.

### Cortical organoid differentiation

Cortical organoids are a promising culture system for studying neurodevelopmental processes (*53*). These organoids at early stages (up to 21 days in vitro) contain large numbers of neural stem and progenitor cells that express SOX2 and Ki67, arranged into neuroepithelial structures called “rosettes.” The organization of these rosettes provides insights into NSC proliferation and survival, which is the primary reason for using organoids. Cortical organoid differentiation was performed as previously described (*52*, *54*). The hESCs were dissociated using Accutase (Sigma-Aldrich) and seeded at 15,000 cells per 96 wells on ultralow attachment U-bottom plates. Cortical organoid differentiation was performed in N2B27 media (50% DMEM/F12, 50% Neuro medium, 1% l-Glutamax, 1% minimum essential medium nonessential amino acids supplemented with 1% N2 supplement, and 2% B27 without vitamin A supplement) (D0 to D21). From days 0 to 5, neural induction was carried out with 10 μM SB431542 (Miltenyi Biotec) and 0.5 μM LDN193189 (Sigma Aldrich), after which neural expansion was promoted from days 6 to 21 with supplementation of EGF (20 ng/ml, Miltenyi Biotec) and FGF2 (20 ng/ml, Miltenyi Biotec).

### Plasmid constructions

The open reading frames of DMTF1 was cloned into PCMV-Tag2B vector by using restriction enzymes EcoRI and HindIII. The pHAGE-DMTF1-V5-GFP was generated using Gibson Assembly kit (New England Biolabs). The pHAGE-DMTF1^∆CDB^-V5-GFP and pHAGE-DMTF1^∆Myb^-V5-GFP mutants were generated using Q5 Site-Directed Mutagenesis Kit (New England Biolabs). The Ss18 promoter sequence was cloned into pGL3-Basic vector, and the Ss18 mutant1 and mutant2 were generated using Q5 Site-Directed Mutagenesis Kit. The primers used for cloning are listed in table S3.

The shRNAs against mouse DMTF1 (shDMTF1#1, TRCN0000077823 and shDMTF1#2, TRCN0000077826) and human DMTF1 (shDMTF1#1, TRCN0000330008 and shDMTF1#2, TRCN0000369279) were purchased from Sigma-Aldrich. The shRNAs against mouse Arid2 and Ss18 were designed using the Broad Institute Genetic Perturbation Platform portal (https://portals.broadinstitute.org/gpp/public/clone/search) and cloned into the pLKO.1 puro vector (Addgene, # 8453). The shRNA against human p53 was provided by C. F. Cheok. The target sequences of shRNAs are listed in table S4.

### Luciferase reporter assay

The Ss18 promoter sequence was cloned into pGL3-Basic vector. The Ss18 promoter-luciferase construct and Renilla plasmids (150 ng:1 ng) were cotransfected into HEK 293T cells. After 48 hours of transfection, the cells were collected to measure the luciferase activity by Dual-Glo luciferase assay system (Promega). The luciferase activity was tested by Varioskan LUX Multimode Microplate Reader (Thermo Fisher Scientific).

### ChIP-seq and ChIP-qPCR

Following procedures previously described in (*55*), the cells were cross-linked with 1% formaldehyde for 10 min at RT. The fixation was stopped by 0.25 M glycine. Cells were lysed using SDS CHIP lysis buffer [1% SDS, 10 mM EDTA, and 50 mM tris-HCl (pH 8)]. The lysate was subjected to sonication for 40 cycles at the Bioruptor sonicator (Diagenode) (30-s ON and 30-s OFF). After sonication, the samples were diluted with ChIP dilution buffer [0.01% SDS, 1% Triton X-100, 1.2 mM EDTA, 16.7 mM tris-HCl (pH 8), and 167 mM NaCl]. After preclear, 10% of the lysates were used as input. The remaining lysates were used for immunoprecipitation with antibodies and protein A/G agarose beads overnight. After immunoprecipitation, the bound protein-DNA complex was washed with the following buffers sequentially to remove nonspecific sequences: low-salt wash buffer [0.1% SDS, 1% Triton X-100, 2 mM EDTA, 20 mM tris-HCl (pH 8), and 150 mM NaCl], high-salt wash buffer [0.1% SDS, 1% Triton X-100, 2 mM EDTA, 20 mM tris-HCl (pH 8), and 500 mM NaCl], LiCl wash buffer [0.25 M LiCl, 1% NP40, 1% deoxycholate, 1 mM EDTA, and 10 mM tris-HCl (pH 8)], and TE wash buffer [10 mM tris-HCl (pH 8) and 1 mM EDTA]. The bound protein-DNA complex was eluted with fresh elution buffer (84 mg of NaHCO_3_, 1 ml 10% SDS, and 9 ml of H_2_O). The eluted samples were then reverse cross-linked with NaCl at 65°C overnight for library preparation or ChIP-qPCR. The library preparation and ChIP-seq were done by Novogene (Singapore). The primers used for ChIP-qPCR are listed in table S5.

### ChIP-seq analysis

Data quality was accessed using FASTQC package (https://bioinformatics.babraham.ac.uk/projects/fastqc/). Paired-end raw sequencing reads were trimmed with TrimGalore (https://bioinformatics.babraham.ac.uk/projects/trim_galore/) with default parameters for paired-end datasets. Cleaned reads were then mapped to mm10 genome obtained from Illumina iGenome website by Bowtie2 (2.4.5) (https://github.com/BenLangmead/bowtie2) with parameters: -N 1 -L 25 --no-mixed --no-discordant. Only uniquely mapped reads with MAPQ ≥10 were kept, and PCR duplicates were removed using samtools (v.1.10) (http://htslib.org/). DMTF1 ChIP-Seq peaks were detected by the “macs2 callpeak” function of MACS2 (*56*) using the parameters: “macs2 callpeak -f BAMPE -g mm -q 0.001 --keep-dup all.” BigWig files were generated using “bamCoverage” function from the package deepTools (*57*) using the parameters: -bs 50 -p 12 –normalizeUsing reads per kilobase million (RPKM) and visualized using IGV Browser (https://igv.org/). DMTF1 ChIP-seq peaks annotations were performed with ChIPseeker (*58*). ChIP-Seq DMTF1-binding regions have been further analyzed to detect notably enriched candidate binding motifs using two independent software as follows: (i) MEME-ChIP (https://meme-suite.org/meme/doc/meme-chip.html) (*59*) and (ii) BaMMmotif (https://bammmotif.mpibpc.mpg.de/) (*60*).

### Public datasets and data analyses

Preprocessed single-cell RNA-seq data for mouse NSC subpopulations from two independent public datasets (*41*, *42*) have been obtained from https://github.com/LKremer/AAV-screening (*41*). The first single cell dataset (*34*) included samples from ventricular-SVZ obtained by pooling data from four 4-month-old mice. The second single cell dataset included the pooled SVZ tissue from four mice of 2 months and eight mice of 22 months of age (*35*). Corresponding Uniform Manifold Approximation and Projection, heatmap plots, and dotplots have been generated using Seurat (v5.2.1) and scCustomize (v3.0.1) packages in R (v4.4.3).

Raw fastq files for public ChIP-Seq datasets for H3K27ac (GSM2406791) (*61*), E2F1 (GSM1908011 and GSM1908010) (*62*), Arid2 (GSM4566876) (*63*), Ss18 (GSM4007594) (*64*), and E2F4 (GSM2040953, GSM2040954, GSM2040961, and GSM2040962) (*65*) have been obtained from NCBI GEO archives (https://ncbi.nlm.nih.gov/gds) and reprocessed as follows. Data quality was accessed using FASTQC package. Further reads preprocessing was performed by Trim Galore. Mapping of ChIP-seq reads was done using bowtie2 against mouse genome mm10 with parameters: “-N 1 -L 25 -U.” Further data processing involved the use of samtools package (“samtools sort,” “rmdup,” and “index” functions). Deeptools suite (*57*) was used to generate bigwig files (“bamCoverage” function) as well as enrichment heatmaps (“computeMatrix” and “plotHeatmap” functions). Chip-seq peaks were obtained using the macs2 (*56*) “macs2 callpeak” function. ChIP-seq peaks comparisons were performed using bedtools (https://bedtools.readthedocs.io/en/latest/).

Processed and global scaling normalized bulk mRNA expression microarray data (Affymetrix Rhesus Macaque Genome Array) from Dentate Gyrus hippocampal regions of young and aged rhesus macaques (*Macaca mulatta*) (*66*) were obtained from GSE11697. The analyzed data included six young (mean age, 7.04 ± 0.62 years) and five old macaques (mean age, 23.3 ± 1.89 years).

Processed tumor and nontumor tissue gene expression and clinical data for patients with glioma from TCGA (https://cancer.gov/tcga), Gravedeel *et al.* (*67*), Lee *et al.* (*68*), and Gill *et al.* (*69*) patients cohorts have been obtained from GlioVis portal (https://gliovis.bioinfo.cnio.es/). R package survminer was used for Kaplan-Meier survival analyses and visualization.

### RNA-seq analysis

Total RNA from mouse NSCs transduced with nontargeting shRNA (shNT), shDMTF1#1 and shDMTF1#2 were isolated using RNeasy Mini or Micro Kit (Qiagen) and sent to Novogene (Singapore) for sequencing analysis. Transcriptomic sequencing (RNA-seq) was performed on the Illumina HiSeq platform according to the standard paired-end protocol. RNA-seq data quality was monitored via FASTQC package (*52*). Paired-end raw sequencing reads were trimmed with Trim Galore with default parameters for paired end data. Cleaned reads were mapped to mm10 mouse reference genome using the RSEM pipeline (v1.3.1) (*70*). The corresponding mouse mm10 gene models were obtained from Illumina iGenome website (https://sapac.support.illumina.com/sequencing/sequencing_software/igenome.html). Differential gene expression analysis was carried out using DESeq2 (v1.16.1) (https://github.com/thelovelab/DESeq2) with default settings. Differentially expressed genes are defined by fold change greater than 1.5-fold difference in expression and *P-*adjusted value ≤0.05 with correction for multiple testing using the Benjamini and Hochberg false discovery rate method. Pathway enrichment analyses were performed using Metascape (https://metascape.org/gp/index.html#/main/step1).

### RNA extraction and qRT-PCR

RNA was extracted with RNeasy Mini or Micro Kit (Qiagen) and then used for first-strand cDNA synthesis with SuperScriptIII Reverse Transcriptase (Invitrogen). Quantitative reverse transcriptase PCR (qRT-PCR) was performed using PowerUp SYBR Green Master Mix (Thermo Fisher Scientific). The relative expression of genes was normalized to housekeeping genes, and each assay was performed in triplicate. The primers used for qRT-PCR are listed in table S6.

### Western blot analysis

Cell pellets were lysed using radioimmunoprecipitation assay buffer (Thermo Fisher Scientific) supplemented with protease inhibitor (Roche) and phosphatase inhibitor (Roche). Protein concentration was determined using DC Protein Assay Kit I (Bio-Rad). Equal amount of protein samples were loaded into SDS–polyacrylamide gel electrophoresis gel and then transferred onto nitrocellulose membranes (Bio-Rad). The membranes were blocked in tris-buffered saline (TBS)–Tween-20 (TBS-T) with 5% skim milk and then incubated with primary antibodies (table S1) overnight at 4°C. The membranes were washed three times in TBS-T before incubating with secondary antibodies for 1 hour at RT. After being washed three times in TBS-T, the membranes were developed using the ChemiDocTM Touch Imaging System (Bio-Rad). Quantification of protein band intensity was performed using ImageJ.

### Histone protein extraction

The cell pellets were resuspended with histone extraction buffer (PBS containing 0.5% Triton X100, 2 mM phenylmethylsulphonyl fluoride, and 0.02% NaN3) at a cell density of 10^7^ cells/1 ml histone extraction buffer. The lysates were put on ice for 10 min with gentle stirring and then centrifuged at 2000 rpm for 10 min at 4°C. The supernatant was removed, and the pellet was washed (washing buffer: PBS containing 0.5% Triton X100) in half the volume of histone extraction buffer and centrifuged at 2000 rpm for 10 min at 4°C. The pellets were resuspended in 0.2 N HCL at a cell density of 4 × 10^7^ cells/1 ml. After incubation at 4°C overnight, the samples were centrifuged at 2000 rpm for 10 min at 4°C. The supernatant was removed, and protein concentration was determined using DC Protein Assay Kit I (Bio-Rad). The samples were analyzed using Western blot analysis.

### Cell cycle analysis

Following procedures previously described in (*71*), the cell pellets were washed with ice-cold 1× PBS and fixed in 70% ethanol at 4°C overnight. Cell pellets were then washed with 1× PBS (ice-cold) and resuspended in propidium iodide (PI, Sigma-Aldrich) staining solution [PI (50 μg/ml), 0.3% Triton X-100, and ribonuclease A (1 mg/ml)]. After incubation at RT for 45 min, the cells were analyzed using Fortessa Cell Analyzer. The cell cycle distribution was analyzed through FlowJo software. The data presented were calculated from three replicates.

### Telomere-FISH

Following procedures previously described in (*72*), the cells were fixed in 4% PFA at RT for 15 min and washed in 1× PBS for 5 min. After fixation, the cells were permeabilized with 0.3% Triton X-100 in 1× PBS (PBS-T) for 15 min. The cells were incubated with V5 (Cell Signaling Technology) primary antibody at 4°C overnight. The cells were washed with 0.3% PBS-T three times and then incubated with goat anti-rabbit immunoglobulin G Alexa Fluor 647 secondary antibody (Invitrogen) at RT for 2 hours in the dark. After washing with 0.3% PBS-T three times, the cells were fixed again with 4% PFA at RT for 10 min. The cells were washed with 1× PBS three times. The fresh hybridization buffer was prepared with 20 mM tris (pH 7.4), 60% formamide, 5% blocking reagent (Roche), and 500 nM telomeric DNA probe (PNABio. F1002 TelC-Cy3) and preheated to 85°C. The cells were prewarmed and incubated in hybridization buffer. The cells were heated for 10 min at 85°C and then placed at RT in the dark for hybridization for 2 hours. After hybridization, the cells were washed with wash solution [2× saline-sodium citrate (SSC) buffer +0.1% Tween-20] for 10 min twice at 55°C and once at RT. The 4′,6-diamidino-2-phenylindole solution was added to the slide. After 10 min, the cells were washed with 2× SSC, 1× SSC, and finally with water for 2 min each. Last, the slide was mounted with Prolong Gold anti-fade (Thermo Fisher Scientific) and cells were observed using confocal (Zeiss, LSM710) with appropriate filters.

### Statistical analyses

All the quantitative data were presented as means ± SDs as described in the figure legends. For computing the statistical significance, the Student’s *t* test was performed using Graph Pad Prism (Version 9.3.1) or Wilcoxon-Mann-Whitney test using Cytel studio (Version 9.0.0). Significance was defined as *P* < 0.05. For the DMTF1 immunofluorescence microscopy analysis shown in Fig. 1B, *n* = 6 mice and the data significance was calculated using the normalized mean of DMTF1 intensity from 15 Nestin^+^ cells per mouse brain. For the BrdU analysis shown in Figs. 1G and 2D and figs. S1E, S3D, and S4C, *n* = 3 biological replicates, and the data significance was calculated using the mean of percentage of BrdU^+^ cells from three to six images per replicate. For the telomere–fluorescence in situ hybridization (FISH) analysis shown in Fig. 1I, *n* = 3 biological replicates and the data significance was calculated using mean of the telomere intensity from 10 cells per replicate. For the EdU labeling assay shown in Fig. 5C, *n* = 3 biological replicates and the data significance was calculated using the mean of EdU intensity from 30 cells per replicate. For the neurosphere formation assay shown in Figs. 2B and 5H, *n* = 6 technical replicates, and the data significance was calculated using the normalized number of neurospheres across the technical replicates in a representative experiment. For the cell viability assays shown in Fig. 5J and fig. S6E, *n* = 6 technical replicates, and the data significance was calculated using normalized luminescence of the technical replicates in a representative experiment. For the cortical organoid experiments shown in Fig. 2 (E to I), *n* = 6 biological replicates, and the data significance was calculated using the indicated measurements of the organoids. For the luciferase assay, *n* = 3 technical replicates, and the data significance was calculated using the normalized dual luciferase ratio of the replicates in a representative experiment. For quantification of Western blot bands, *n* = 3 biological replicates, and the data significance was calculated using normalized band intensity of the indicated proteins across the replicates. For the qPCR and ChIP-qPCR assays, *n* = 3 technical replicates, and the data significance was calculated using the normalized values across the replicates in a representative experiment.

## References

[1] M. P. Mattson, T. Magnus, Ageing and neuronal vulnerability. Nat. Rev. Neurosci. 7, 278–294 (2006). 16552414 10.1038/nrn1886 PMC3710114

[2] A. M. Nicaise, C. M. Willis, S. J. Crocker, S. Pluchino, Stem cells of the aging brain. Front. Aging Neurosci. 12, 247 (2020). 32848716 10.3389/fnagi.2020.00247 PMC7426063

[3] B. A. Yankner, T. Lu, P. Loerch, The aging brain. Annu. Rev. Pathol. 3, 41–66 (2008). 18039130 10.1146/annurev.pathmechdis.2.010506.092044

[4] C. Lopez-Otin, M. A. Blasco, L. Partridge, M. Serrano, G. Kroemer, Hallmarks of aging: An expanding universe. Cell 186, 243–278 (2023). 36599349 10.1016/j.cell.2022.11.001

[5] M. Jaskelioff, F. L. Muller, J. H. Paik, E. Thomas, S. Jiang, A. C. Adams, E. Sahin, M. Kost-Alimova, A. Protopopov, J. Cadiñanos, J. W. Horner, E. Maratos-Flier, R. A. DePinho, Telomerase reactivation reverses tissue degeneration in aged telomerase-deficient mice. Nature 469, 102–106 (2011). 21113150 10.1038/nature09603 PMC3057569

[6] H. W. Lee, M. A. Blasco, G. J. Gottlieb, J. W. Horner, C. W. Greider, R. A. DePinho, Essential role of mouse telomerase in highly proliferative organs. Nature 392, 569–574 (1998). 9560153 10.1038/33345

[7] E. Sahin, R. A. DePinho, Linking functional decline of telomeres, mitochondria and stem cells during ageing. Nature 464, 520–528 (2010). 20336134 10.1038/nature08982 PMC3733214

[8] K. Whittemore, A. Derevyanko, P. Martinez, R. Serrano, M. Pumarola, F. Bosch, M. A. Blasco, Telomerase gene therapy ameliorates the effects of neurodegeneration associated to short telomeres in mice. Aging 11, 2916–2948 (2019). 31140977 10.18632/aging.101982 PMC6555470

[9] D. S. Leeman, K. Hebestreit, T. Ruetz, A. E. Webb, A. McKay, E. A. Pollina, B. Dulken, X. A. Zhao, R. W. Yeo, T. T. Ho, S. Mahmoudi, K. Devarajan, E. Passegue, T. A. Rando, J. Frydman, A. Brunet, Lysosome activation clears aggregates and enhances quiescent neural stem cell activation during aging. Science 359, 1277–1283 (2018). 29590078 10.1126/science.aag3048 PMC5915358

[10] P. N. Negredo, R. W. Yeo, A. Brunet, Aging and rejuvenation of neural stem cells and their niches. Cell Stem Cell 27, 202–223 (2020). 32726579 10.1016/j.stem.2020.07.002 PMC7415725

[11] R. W. Yeo, O. Y. Zhou, B. L. Zhong, E. D. Sun, P. N. Negredo, S. Nair, M. Sharmin, T. J. Ruetz, M. Wilson, A. Kundaje, A. R. Dunn, A. Brunet, Chromatin accessibility dynamics of neurogenic niche cells reveal defects in neural stem cell adhesion and migration during aging. Nat. Aging 3, 866–893 (2023). 37443352 10.1038/s43587-023-00449-3 PMC10353944

[12] E. A. Fry, K. Inoue, c-MYB and DMTF1 in Cancer. Cancer Invest. 37, 46–65 (2019). 30599775 10.1080/07357907.2018.1550090 PMC6431554

[13] A. Mallakin, T. Sugiyama, P. Taneja, L. A. Matise, D. P. Frazier, M. Choudhary, G. A. Hawkins, R. B. D'Agostino Jr., M. C. Willingham, K. Inoue, Mutually exclusive inactivation of DMP1 and ARF/p53 in lung cancer. Cancer Cell 12, 381–394 (2007). 17936562 10.1016/j.ccr.2007.08.034 PMC2239345

[14] K. Inoue, F. Zindy, D. H. Randle, J. E. Rehg, C. J. Sherr, Dmp1 is haplo-insufficient for tumor suppression and modifies the frequencies of Arf and p53 mutations in Myc-induced lymphomas. Genes Dev. 15, 2934–2939 (2001). 11711428 10.1101/gad.929901 PMC312824

[15] P. Taneja, D. Maglic, F. Kai, T. Sugiyama, R. D. Kendig, D. P. Frazier, M. C. Willingham, K. Inoue, Critical roles of DMP1 in human epidermal growth factor receptor 2/neu-Arf-p53 signaling and breast cancer development. Cancer Res. 70, 9084–9094 (2010). 21062982 10.1158/0008-5472.CAN-10-0159 PMC3073839

[16] K. Inoue, M. F. Roussel, C. J. Sherr, Induction of ARF tumor suppressor gene expression and cell cycle arrest by transcription factor DMP1. Proc. Natl. Acad. Sci. U.S.A. 96, 3993–3998 (1999). 10097151 10.1073/pnas.96.7.3993 PMC22408

[17] D. P. Frazier, R. D. Kendig, F. Kai, D. Maglic, T. Sugiyama, R. L. Morgan, E. A. Fry, S. J. Lagedrost, G. C. Sui, K. Inoue, Dmp1 physically interacts with p53 and positively regulates p53's stability, nuclear localization, and function. Cancer Res. 72, 1740–1750 (2012). 22331460 10.1158/0008-5472.CAN-11-2410 PMC3319807

[18] K. Inoue, R. R. Wen, J. E. Rehg, M. Adachi, J. L. Cleveland, M. F. Roussel, C. J. Sherr, Disruption of the ARF transcriptional activator DMP1 facilitates cell immortalization, Ras transformation, and tumorigenesis. Genes Dev. 14, 1797–1809 (2000). 10898794 PMC316790

[19] M. Kobayashi, E. F. Srour, Regulation of murine hematopoietic stem cell quiescence by Dmtf1. Blood 118, 6562–6571 (2011). 22039255 10.1182/blood-2011-05-349084 PMC3242718

[20] C. Kadoch, G. R. Crabtree, Mammalian SWI/SNF chromatin remodeling complexes and cancer: Mechanistic insights gained from human genomics. Sci. Adv. 1, e1500447 (2015). 26601204 10.1126/sciadv.1500447 PMC4640607

[21] P. Mittal, C. W. M. Roberts, The SWI/SNF complex in cancer - Biology, biomarkers and therapy. Nat. Rev. Clin. Oncol. 17, 435–448 (2020). 32303701 10.1038/s41571-020-0357-3 PMC8723792

[22] C. Kadoch, D. C. Hargreaves, C. Hodges, L. Elias, L. Ho, J. Ranish, G. R. Crabtree, Proteomic and bioinformatic analysis of mammalian SWI/SNF complexes identifies extensive roles in human malignancy. Nat. Genet. 45, 592–601 (2013). 23644491 10.1038/ng.2628 PMC3667980

[23] L. Ho, E. L. Miller, J. L. Ronan, W. Q. Ho, R. Jothi, G. R. Crabtree, esBAF facilitates pluripotency by conditioning the genome for LIF/STAT3 signalling and by regulating polycomb function. Nat. Cell Biol. 13, 903–913 (2011). 21785422 10.1038/ncb2285 PMC3155811

[24] B. G. Wilson, X. Wang, X. H. Shen, E. S. McKenna, M. E. Lemieux, Y. J. Cho, E. C. Koellhoffer, S. L. Pomeroy, S. H. Orkin, C. W. M. Roberts, Epigenetic antagonism between polycomb and SWI/SNF complexes during oncogenic transformation. Cancer Cell 18, 316–328 (2010). 20951942 10.1016/j.ccr.2010.09.006 PMC2957473

[25] B. H. Alver, K. H. Kim, P. Lu, X. F. Wang, H. E. Manchester, W. S. Wang, J. R. Haswell, P. J. Park, C. W. M. Roberts, The SWI/SNF chromatin remodelling complex is required for maintenance of lineage specific enhancers. Nat. Commun. 8, 14648 (2017). 28262751 10.1038/ncomms14648 PMC5343482

[26] H. Kang, K. R. Cui, K. J. Zhao, BRG1 controls the activity of the retinoblastoma protein via regulation of p21CIP1/WAF1/SDI. Mol. Cell. Biol. 24, 1188–1199 (2004). 14729964 10.1128/MCB.24.3.1188-1199.2004 PMC321457

[27] N. G. Nagl Jr., D. R. Zweitzig, B. Thimmapaya, G. R. Beck Jr., E. Moran, The c-myc gene is a direct target of mammalian SWI/SNF-related complexes during differentiation-associated cell cycle arrest. Cancer Res. 66, 1289–1293 (2006). 16452181 10.1158/0008-5472.CAN-05-3427

[28] S. Matsumoto, F. Banine, J. Struve, R. B. Xing, C. Adams, Y. Liu, D. Metzger, P. Chambon, M. S. Rao, L. S. Sherman, Brg1 is required for murine neural stem cell maintenance and gliogenesis. Dev. Biol. 289, 372–383 (2006). 16330018 10.1016/j.ydbio.2005.10.044

[29] S. Matsumoto, F. Banine, K. Feistel, S. Foster, R. B. Xing, J. Struve, L. S. Sherman, Brg1 directly regulates Olig2 transcription and is required for oligodendrocyte progenitor cell specification. Dev. Biol. 413, 173–187 (2016). 27067865 10.1016/j.ydbio.2016.04.003 PMC4851915

[30] B. T. Staahl, J. Tang, W. Wu, A. Sun, A. D. Gitler, A. S. Yoo, G. R. Crabtree, Kinetic analysis of npBAF to nBAF switching reveals exchange of SS18 with CREST and integration with neural developmental pathways. J. Neurosci. 33, 10348–10361 (2013). 23785148 10.1523/JNEUROSCI.1258-13.2013 PMC3685834

[31] S. M. G. Braun, R. Petrova, J. Tang, A. Krokhotin, E. L. Miller, Y. T. Tang, G. Panagiotakos, G. R. Crabtree, BAF subunit switching regulates chromatin accessibility to control cell cycle exit in the developing mammalian cortex. Genes Dev. 35, 335–353 (2021). 33602870 10.1101/gad.342345.120 PMC7919417

[32] O. Păun, Y. X. Tan, H. Patel, S. Strohbuecker, A. Ghanate, C. Cobolli-Gigli, M. L. Sopena, L. Gerontogianni, R. Goldstone, S.-L. Ang, F. Guillemot, C. Dias, Pioneer factor ASCL1 cooperates with the mSWI/SNF complex at distal regulatory elements to regulate human neural differentiation. Genes Dev. 37, 218–242 (2023). 36931659 10.1101/gad.350269.122 PMC10111863

[33] S. E. Keegan, J. Haskins, A. J. Simmonds, S. C. Hughes, A chromatin remodelling SWI/SNF subunit, Snr1, regulates neural stem cell determination and differentiation. Development 150, dev201484 (2023). 37294080 10.1242/dev.201484 PMC10323235

[34] V. K. Chmykhalo, R. V. Deev, A. T. Tokarev, Y. A. Polunina, L. Xue, Y. V. Shidlovskii, SWI/SNF complex connects signaling and epigenetic state in cells of nervous system. Mol. Neurobiol. 62, 1536–1557 (2025). 39002058 10.1007/s12035-024-04355-6

[35] C. C. Liu, D. L. Ma, T. D. Yan, X. B. Fan, Z. Y. Poon, L. F. Poon, S. A. Goh, S. G. Rozen, W. Y. K. Hwang, V. Tergaonkar, P. Tan, S. Ghosh, D. M. Virshup, E. L. K. Goh, S. Li, Distinct responses of stem cells to telomere uncapping—A potential strategy to improve the safety of cell therapy. Stem Cells 34, 2471–2484 (2016). 27299710 10.1002/stem.2431

[36] L. Chin, S. E. Artandi, Q. Shen, A. Tam, S. L. Lee, G. J. Gottlieb, C. W. Greider, R. A. DePinho, p53 deficiency rescues the adverse effects of telomere loss and cooperates with telomere dysfunction to accelerate carcinogenesis. Cell 97, 527–538 (1999). 10338216 10.1016/s0092-8674(00)80762-x

[37] M. Fischer, Census and evaluation of p53 target genes. Oncogene 36, 3943–3956 (2017). 28288132 10.1038/onc.2016.502 PMC5511239

[38] H. Hirai, C. J. Sherr, Interaction of D-type cyclins with a novel myb-like transcription factor, DMP1. Mol. Cell. Biol. 16, 6457–6467 (1996). 8887674 10.1128/mcb.16.11.6457 PMC231647

[39] K. Inoue, A. Mallakin, D. P. Frazier, Dmp1 and tumor suppression. Oncogene 26, 4329–4335 (2007). 17237816 10.1038/sj.onc.1210226 PMC2077852

[40] M. A. Marqués-Torrejón, C. A. C. Williams, B. Southgate, N. Alfazema, M. P. Clements, C. Garcia-Diaz, C. Blin, N. Arranz-Emparan, J. Fraser, N. Gammoh, S. Parrinello, S. M. Pollard, LRIG1 is a gatekeeper to exit from quiescence in adult neural stem cells. Nat. Commun. 12, 2594 (2021). 33972529 10.1038/s41467-021-22813-w PMC8110534

[41] L. P. M. Kremer, S. Cerrizuela, S. Dehler, T. Stiehl, J. Weinmann, H. Abendroth, S. Kleber, A. Laure, J. El Andari, S. Anders, A. Marciniak-Czochra, D. Grimm, A. Martin-Villalba, High throughput screening of novel AAV capsids identifies variants for transduction of adult NSCs within the subventricular zone. Mol. Ther. Methods Clin. Dev. 23, 33–50 (2021). 34553001 10.1016/j.omtm.2021.07.001 PMC8427210

[42] G. Kalamakis, D. Brüne, S. Ravichandran, J. Bolz, W. Q. Fan, F. Ziebell, T. Stiehl, F. Catalá-Martinez, J. Kupke, S. Zhao, E. Llorens-Bobadilla, K. Bauer, S. Limpert, B. Berger, U. Christen, P. Schmezer, J. P. Mallm, B. Berninger, S. Anders, A. del Sol, A. Marciniak-Czochra, A. Martin-Villalba, Quiescence modulates stem cell maintenance and regenerative capacity in the aging brain. Cell 176, 1407–1419.e14 (2019). 30827680 10.1016/j.cell.2019.01.040

[43] J. Hsu, J. Arand, A. Chaikovsky, N. A. Mooney, J. Demeter, C. M. Brison, R. Oliverio, H. Vogel, S. M. Rubin, P. K. Jackson, J. Sage, E2F4 regulates transcriptional activation in mouse embryonic stem cells independently of the RB family. Nat. Commun. 10, 2939 (2019). 31270324 10.1038/s41467-019-10901-x PMC6610666

[44] J. W. Wang, Q. Sun, Y. Morita, H. Jiang, A. Gross, A. Lechel, K. Hildner, L. M. Guachalla, A. Gompf, D. Hartmann, A. Schambach, T. Wuestefeld, D. Dauch, H. Schrezenmeier, W. K. Hofmann, H. Nakauchi, Z. Y. Ju, H. A. Kestler, L. Zender, K. L. Rudolph, A differentiation checkpoint limits hematopoietic stem cell self-renewal in response to DNA damage. Cell 148, 1001–1014 (2012). 22385964 10.1016/j.cell.2012.01.040

[45] L. Mahendrawada, L. Warfield, R. Donczew, S. Hahn, Low overlap of transcription factor DNA binding and regulatory targets. Nature 642, 796–804 (2025). 40240607 10.1038/s41586-025-08916-0 PMC12882699

[46] K. Inoue, C. J. Sherr, L. H. Shapiro, Regulation of the CD13/aminopeptidase N gene by DMP1, a transcription factor antagonized by D-type cyclins. J. Biol. Chem. 273, 29188–29194 (1998). 9786929 10.1074/jbc.273.44.29188

[47] S. Carcamo, C. B. Nguyen, E. Grossi, D. Filipescu, A. Alpsoy, A. Dhiman, D. Sun, S. Narang, J. Imig, T. C. Martin, R. Parsons, I. Aifantis, A. Tsirigos, J. A. Aguirre-Ghiso, E. C. Dykhuizen, D. Hasson, E. Bernstein, Altered BAF occupancy and transcription factor dynamics in PBAF-deficient melanoma. Cell Rep. 39, 110637 (2022). 35385731 10.1016/j.celrep.2022.110637 PMC9013128

[48] M. Fischer, A. E. Schade, T. B. Branigan, G. A. Müller, J. A. DeCaprio, Coordinating gene expression during the cell cycle. Trends Biochem. Sci. 47, 1009–1022 (2022). 35835684 10.1016/j.tibs.2022.06.007

[49] M. Fischer, P. Grossmann, M. Padi, J. A. DeCaprio, Integration of TP53, DREAM, MMB-FOXM1 and RB-E2F target gene analyses identifies cell cycle gene regulatory networks. Nucleic Acids Res. 44, 6070–6086 (2016). 27280975 10.1093/nar/gkw523 PMC4994865

[50] M. Jeitany, D. Bakhos-Douaihy, D. C. Silvestre, J. R. Pineda, N. Ugolin, A. Moussa, L. R. Gauthier, D. Busso, M. P. Junier, H. Chneiweiss, S. Chevillard, C. Desmaze, F. D. Boussin, Opposite effects of GCN5 and PCAF knockdowns on the alternative mechanism of telomere maintenance. Oncotarget 8, 26269–26280 (2017). 28412741 10.18632/oncotarget.15447 PMC5432255

[51] F. Li, Z. Deng, L. Zhang, C. Z. Wu, Y. Jin, I. Hwang, O. Vladimirova, L. B. Xu, L. Yang, B. Lu, J. Dheekollu, J. Y. Li, H. Feng, J. Hu, C. R. Vakoc, H. Q. Ying, J. Paik, P. M. Lieberman, H. W. Zheng, ATRX loss induces telomere dysfunction and necessitates induction of alternative lengthening of telomeres during human cell immortalization. EMBO J. 38, e96659 (2019). 31454099 10.15252/embj.201796659 PMC6769380

[52] J. Feng, Y. H. Chuah, Y. Liang, N. O. Cipta, Y. Zeng, T. Warrier, G. Elfar, J. Yoon, O. V. Grinchuk, E. X. Y. Tay, K. Z. Lok, Z. Q. Zheng, Z. J. Khong, Z. S. Chong, J. Teo, E. M. Sanford, C. J. Y. Neo, H. Y. Chiu, J. Y. Leung, L. C. Wang, Y. T. Lim, T. Zhao, R. M. Sobota, K. C. Crasta, V. Tergaonkar, R. Taneja, S. Y. Ng, C. F. Cheok, S. C. Ling, Y. H. Loh, D. S. T. Ong, PHF2 regulates genome topology and DNA replication in neural stem cells via cohesin. Nucleic Acids Res. 52, 7063–7080 (2024). 38808662 10.1093/nar/gkae457 PMC11229317

[53] M. A. Lancaster, M. Renner, C. A. Martin, D. Wenzel, L. S. Bicknell, M. E. Hurles, T. Homfray, J. M. Penninger, A. P. Jackson, J. A. Knoblich, Cerebral organoids model human brain development and microcephaly. Nature 501, 373–379 (2013). 23995685 10.1038/nature12517 PMC3817409

[54] A. M. Pasca, S. A. Sloan, L. E. Clarke, Y. Tian, C. D. Makinson, N. Huber, C. H. Kim, J. Y. Park, N. A. O'Rourke, K. D. Nguyen, S. J. Smith, J. R. Huguenard, D. H. Geschwind, B. A. Barres, S. P. Pasca, Functional cortical neurons and astrocytes from human pluripotent stem cells in 3D culture. Nat. Methods 12, 671–678 (2015). 26005811 10.1038/nmeth.3415 PMC4489980

[55] B. W. L. Lee, Y. H. Chuah, J. Yoon, O. V. Grinchuk, Y. Liang, J. L. Hirpara, Y. Shen, L. C. Wang, Y. T. Lim, T. Zhao, R. M. Sobota, T. T. Yeo, A. L. A. Wong, K. Teo, V. D. W. Nga, B. W. Q. Tan, T. Suda, T. B. Toh, S. Pervaiz, Z. Lin, D. S. T. Ong, METTL8 links mt-tRNA m^3^C modification to the HIF1α/RTK/Akt axis to sustain GBM stemness and tumorigenicity. Cell Death Dis. 15, 338 (2024). 38744809 10.1038/s41419-024-06718-2 PMC11093979

[56] Y. Zhang, T. Liu, C. A. Meyer, J. Eeckhoute, D. S. Johnson, B. E. Bernstein, C. Nussbaum, R. M. Myers, M. Brown, W. Li, X. S. Liu, Model-based analysis of ChIP-seq (MACS). Genome Biol. 9, R137 (2008). 18798982 10.1186/gb-2008-9-9-r137 PMC2592715

[57] F. Ramírez, F. Dündar, S. Diehl, B. A. Grüning, T. Manke, deepTools: A flexible platform for exploring deep-sequencing data. Nucleic Acids Res. 42, W187–W191 (2014). 24799436 10.1093/nar/gku365 PMC4086134

[58] G. C. Yu, L.-G. Wang, Q.-Y. He, ChIPseeker: An R/Bioconductor package for ChIP peak annotation, comparison and visualization. Bioinformatics 31, 2382–2383 (2015). 25765347 10.1093/bioinformatics/btv145

[59] P. Machanick, T. L. Bailey, MEME-ChIP: Motif analysis of large DNA datasets. Bioinformatics 27, 1696–1697 (2011). 21486936 10.1093/bioinformatics/btr189 PMC3106185

[60] A. Kiesel, C. Roth, W. W. Ge, M. Wess, M. Meier, J. Söding, The BaMM web server for de-novo motif discovery and regulatory sequence analysis. Nucleic Acids Res. 46, W215–W220 (2018). 29846656 10.1093/nar/gky431 PMC6030882

[61] J. A. Bertolini, R. Favaro, Y. F. Zhu, M. Pagin, C. Y. Ngan, C. H. Wong, H. Tjong, M. W. Vermunt, B. Martynoga, C. Barone, J. Mariani, M. J. Cardozo, N. Tabanera, F. Zambelli, S. Mercurio, S. Ottolenghi, P. Robson, M. P. Creyghton, P. Bovolenta, G. Pavesi, F. Guillemot, S. K. Nicolis, C.-L. Wei, Mapping the global chromatin connectivity network for Sox2 function in neural stem cell maintenance. Cell Stem Cell 24, 462–476.e6 (2019). 30849367 10.1016/j.stem.2019.02.004 PMC6506828

[62] P. D. Denechaud, I. C. Lopez-Mejia, A. Giralt, Q. W. Lai, E. Blanchet, B. Delacuisine, B. N. Nicolay, N. J. Dyson, C. Bonner, F. Pattou, J. S. Annicotte, L. Fajas, E2F1 mediates sustained lipogenesis and contributes to hepatic steatosis. J. Clin. Investig. 126, 137–150 (2016). 26619117 10.1172/JCI81542 PMC4701565

[63] Y.-K. Park, J.-E. Lee, Z. J. Yan, K. McKernan, T. O'Haren, W. D. Wang, W. Q. Peng, K. Ge, Interplay of BAF and MLL4 promotes cell type-specific enhancer activation. Nat. Commun. 12, 1630 (2021). 33712604 10.1038/s41467-021-21893-y PMC7955098

[64] J. Q. Kuang, Z. W. Zhai, P. L. Li, R. N. Shi, W. J. Guo, Y. X. Yao, J. Guo, G. Q. Zhao, J. P. He, S. Y. Xu, C. M. Wu, S. Y. Yu, C. H. Zhou, L. L. Wu, Y. Qin, B. M. Cai, W. Li, Z. C. Wu, X. X. Li, S. L. Chu, T. T. Yang, B. Wang, S. T. Cao, D. W. Li, X. F. Zhang, J. K. Chen, J. Liu, D. Q. Pei, SS18 regulates pluripotent-somatic transition through phase separation. Nat. Commun. 12, 4090 (2021). 34215745 10.1038/s41467-021-24373-5 PMC8253816

[65] X. Sun, J.-C. Chuang, M. Kanchwala, L. W. Wu, C. Celen, L. Li, H. Q. Liang, S. Y. Zhang, T. Maples, L. H. Nguyen, S. C. Wang, R. A. J. Signer, M. Sorouri, I. Nassour, X. Liu, J. Xu, M. Wu, Y. Zhao, Y. C. Kuo, Z. Wang, C. Xing, H. Zhu, Suppression of the SWI/SNF component Arid1a promotes mammalian regeneration. Cell Stem Cell 18, 456–466 (2016). 27044474 10.1016/j.stem.2016.03.001 PMC4826298

[66] E. M. Blalock, R. Grondin, K. C. Chen, O. Thibault, V. Thibault, J. D. Pandya, A. Dowling, Z. M. Zhang, P. Sullivan, N. M. Porter, P. W. Landfield, Aging-related gene expression in hippocampus proper compared with dentate gyrus is selectively associated with metabolic syndrome variables in Rhesus monkeys. J. Neurosci. 30, 6058–6071 (2010). 20427664 10.1523/JNEUROSCI.3956-09.2010 PMC3155249

[67] L. A. M. Gravendeel, M. C. M. Kouwenhoven, O. Gevaert, J. J. de Rooi, A. P. Stubbs, J. E. Duijm, A. Daemen, F. E. Bleeker, L. B. C. Bralten, N. K. Kloosterhof, B. De Moor, P. H. C. Eilers, P. J. van der Spek, J. M. Kros, P. Smitt, M. J. van den Bent, P. J. French, Intrinsic gene expression profiles of gliomas are a better predictor of survival than histology. Cancer Res. 69, 9065–9072 (2009). 19920198 10.1158/0008-5472.CAN-09-2307

[68] Y. Lee, A. C. Scheck, T. F. Cloughesy, A. Lai, J. Dong, H. K. Farooqi, L. M. Liau, S. Horvath, P. S. Mischel, S. F. Nelson, Gene expression analysis of glioblastomas identifies the major molecular basis for the prognostic benefit of younger age. BMC Med. Genomics 1, 52 (2008). 18940004 10.1186/1755-8794-1-52 PMC2596165

[69] B. J. Gill, D. J. Pisapia, H. R. Malone, H. Goldstein, L. Lei, A. Sonabend, J. Yun, J. Samanamud, J. S. Sims, M. Banu, A. Dovas, A. F. Teich, S. A. Sheth, G. M. McKhann, M. B. Sisti, J. N. Bruce, P. A. Sims, P. Canoll, MRI-localized biopsies reveal subtype-specific differences in molecular and cellular composition at the margins of glioblastoma. Proc. Natl. Acad. Sci. U.S.A. 111, 12550–12555 (2014). 25114226 10.1073/pnas.1405839111 PMC4151734

[70] B. Li, C. N. Dewey, RSEM: Accurate transcript quantification from RNA-Seq data with or without a reference genome. BMC Bioinformatics 12, 323 (2011). 21816040 10.1186/1471-2105-12-323 PMC3163565

[71] Y. H. Chuah, E. X. Y. Tay, O. V. Grinchuk, J. Yoon, J. Feng, S. Kannan, M. Robert, R. Jakhar, Y. Liang, B. W. L. Lee, L. C. Wang, Y. T. Lim, T. Zhao, R. M. Sobota, G. Lu, B. C. Low, K. C. Crasta, C. S. Verma, Z. Lin, D. S. T. Ong, CAMK2D serves as a molecular scaffold for RNF8-MAD2 complex to induce mitotic checkpoint in glioma. Cell Death Differ. 30, 1973–1987 (2023). 37468549 10.1038/s41418-023-01192-3 PMC10406836

[72] J. Harley, M. M. Santosa, C. Y. Ng, O. V. Grinchuk, J. H. Hor, Y. Liang, V. J. Lim, W. W. Tee, D. S. T. Ong, S. Y. Ng, Telomere shortening induces aging-associated phenotypes in hiPSC-derived neurons and astrocytes. Biogerontology 25, 341–360 (2024). 37987889 10.1007/s10522-023-10076-5 PMC10998800

