# Supplementary material for: DMTF1 up-regulation rescues proliferation defect of telomere dysfunctional neural stem cells via the SWI/SNF-E2F axis
Source: Sci Adv. 2026 Jan 2;12(1):eady5905. doi: 10.1126/sciadv.ady5905 (PMC12758551; doi:10.1126/sciadv.ady5905)
Supplement: Supplementary file 1 — Figs. S1 to S8 Tables S1 to S6 [file sciadv.ady5905_sm.pdf]

Science Advances
AAAS

Supplementary Materials for

DMTF1 up-regulation rescues proliferation defect of telomere dysfunctional neural stem cells via the SWI/SNF-E2F axis

Yajing Liang *et al.*

Corresponding author: Derrick Sek Tong Ong, phsostd@nus.edu.sg

Sci. Adv. **12**, eady5905 (2026)
DOI: 10.1126/sciadv.ady5905

This PDF file includes:

Figs. S1 to S8
Tables S1 to S6

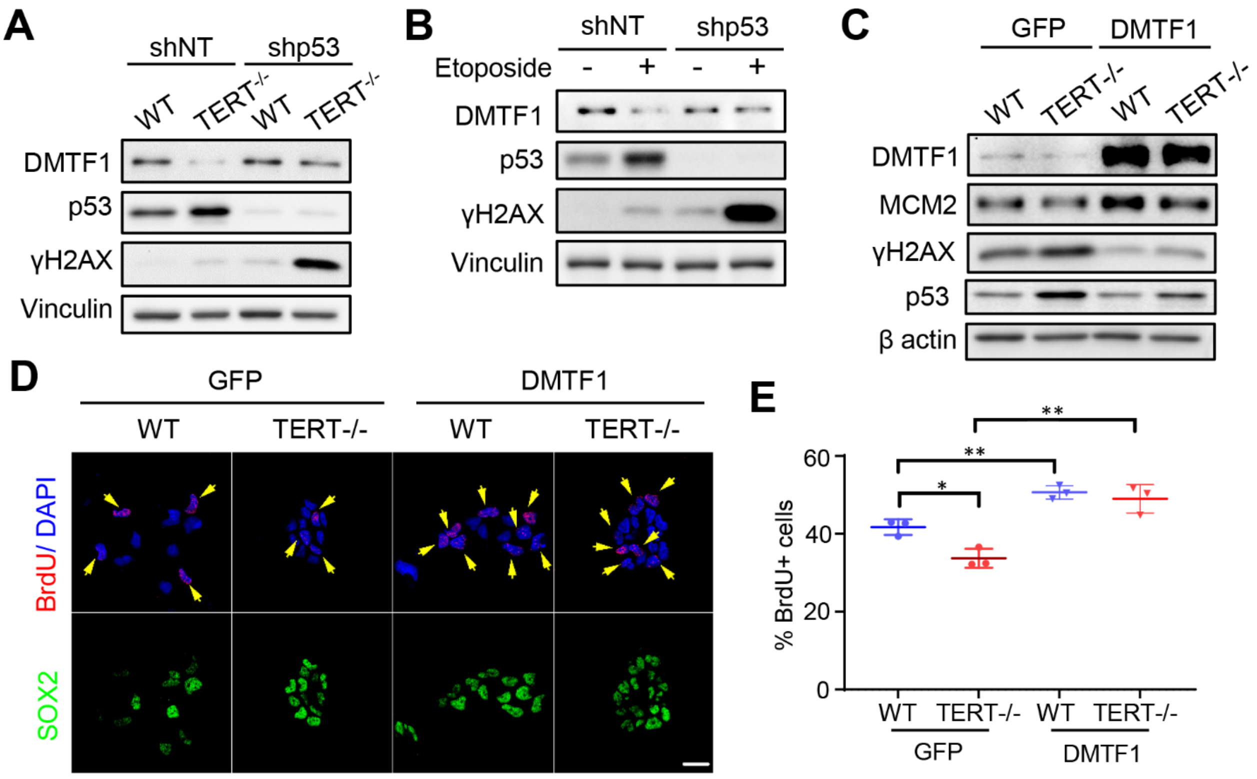

Fig. S1. DMTF1 upregulation rescues proliferation defect of telomere dysfunctional human NSCs *in vitro*. (**A**) Western blot analysis of DMTF1, p53 and γH2AX levels in WT and TERT KO hNPCs, with or without p53 KD. Vinculin serves as the loading control. (**B**) Western blot analysis of DMTF1, p53 and γH2AX levels in etoposide-treated hNPCs, with or without p53 KD. Vinculin serves as the loading control. (**C**) Western blot analysis of DMTF1, MCM2, γH2AX and p53 in WT and TERT KO hNPCs, with or without DMTF1 overexpression. β-actin serves as the loading control. (**D & E**) Representative images (**D**) and quantification (**E**) of $BrdU^+$ cells in WT and TERT KO hNPCs, with or without DMTF1 overexpression. Scale bar: 20μm. (n=3 replicates, 3 fields/images per replicate) (mean ± SD). **P* <0.05, ***P* <0.01.

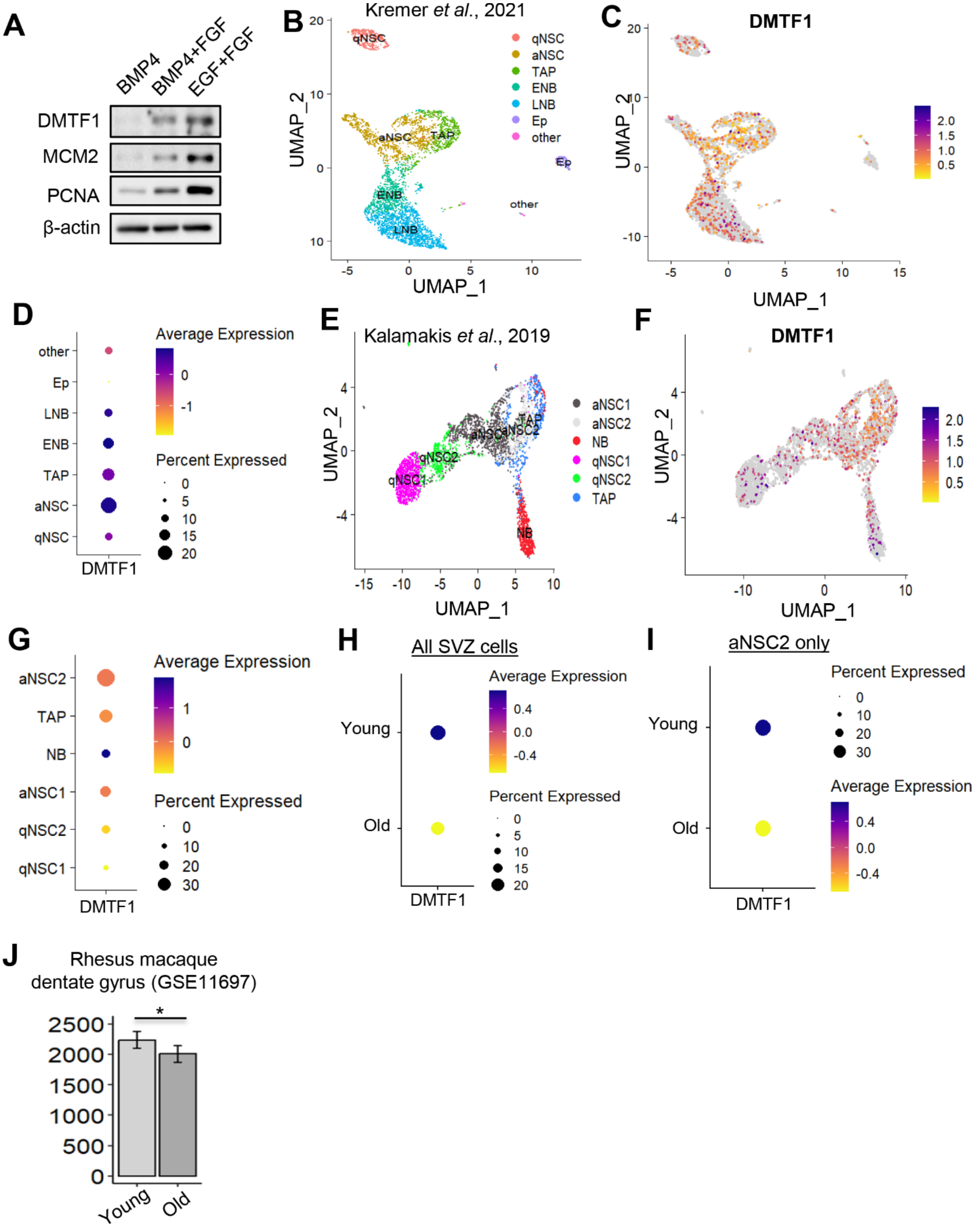

Fig. S2. High DMTF1 expression correlates with NSC proliferation. (**A**) Western blot analysis of DMTF1, MCM2 and PCNA levels in mouse NSCs that are cultured with the indicated growth factors. β-actin serves as the loading control. (**B-I**) Analysis of DMTF1 expression in two publicly

available single-cell RNA-Seq datasets. (**B-D**) UMAP plot, DMTF1 heatmap and dot plot generated using single-cell RNA-Seq data from 4 months old mice (n=4) (*41*). *aNSC*, activated NSC. *NB*, neuroblasts. *qNSC*: quiescent NSC. *aNSC*, activated NSC. *TAP*, transit amplifying progenitor. *ENB*, early neuroblasts. *LNB*, late neuroblasts. *EP*, ependymal cells. (**E-I)** UMAP plot, DMTF1 heatmap and dot plot generated using single-cell RNA-Seq data from young (2 months old, n=4) and old (22 months old, n=8) mice (*42*). *qNSC*: quiescent NSC. *TAP*, transit amplifying progenitor. (**J**) Analysis of DMTF1 expression in the dentate gyrus hippocampal regions of young (mean age 7.04 ±0.62 years, n=6) and old (mean age 23.3±1.89 years, n=5) *Macaca mulatta*. *: $p<0.05$, two-sided t-test.

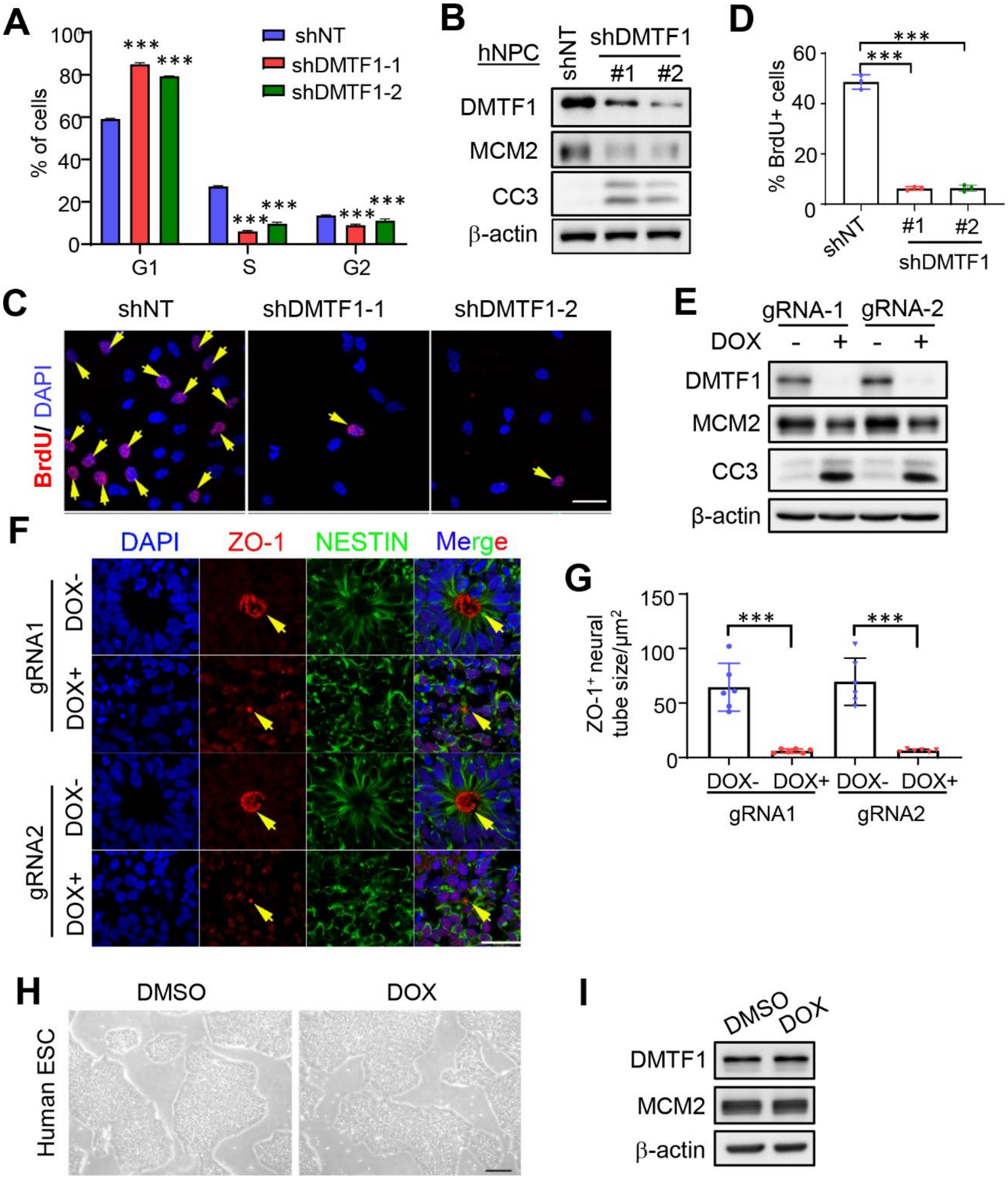

Fig. S3. DMTF1 loss drastically impairs mouse and human NSC proliferation *in vitro*. (**A**) Cell cycle analysis of mouse NSCs upon DMTF1 KD (n=3 replicates) (mean ± SD). ***P <0.001. (**B**) Western blot analysis of DMTF1, MCM2, and CC3 levels in DMTF1 depleted hNPC. β-actin serves as the loading control. (**C & D**) Representative images (**C**) and quantification (**D**) of BrdU^{+} cells in DMTF1 depleted hNPC. Scale bar: 20μm. (n=3 replicates, 3 fields/images per replicate) (mean ± SD). ****P* <0.001. (**E**) Western blot analysis of DMTF1, MCM2, and CC3 levels in DMTF1 KO cortical organoids. β-actin serves as the loading control. (**F**) Representative images of ZO-1 and NESTIN in DMTF1 intact and KO cortical organoids. Scale bar: 20μm. Yellow arrows indicate ZO-1^{+} neuroepithelial structures. (**G**) Quantification of ZO-1^{+} neural tube size in

cortical organoids upon DMTF1 KO (n=6 organoids) (mean ± SD). ****P* <0.001. (**H**) Representative brightfield images of hESC with or without DOX treatment. Scale bar: 200µm. (**I**) Western blot analysis of DMTF1 and MCM2 levels in hESC with or without DOX treatment. β-actin serves as the loading control.

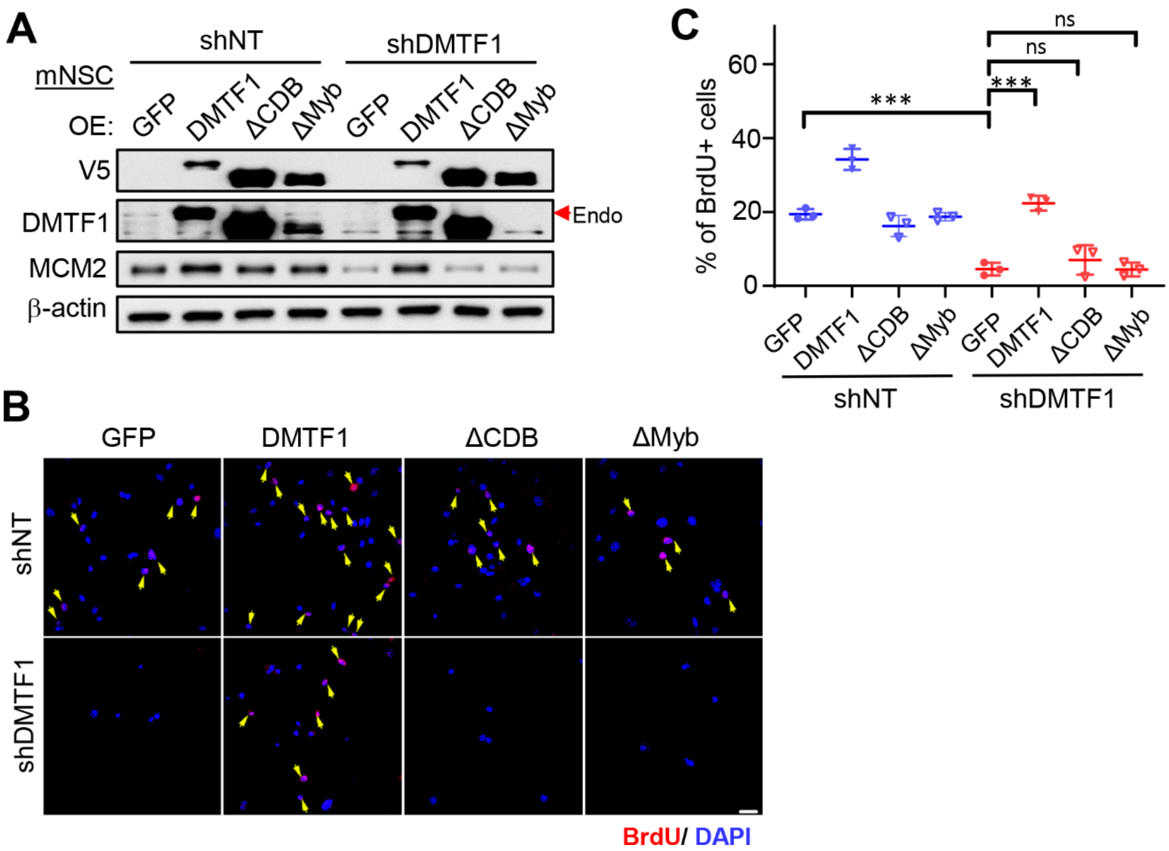

Fig. S4. Validating the functionality of V5-DMTF1 for V5 antibody-mediated ChIP-Seq analysis. (**A**) Western blot analysis of V5, DMTF1 and MCM2 in DMTF1 KD mouse NSCs, with or without overexpression of V5-tagged wildtype, ΔCDB or ΔMyb DMTF1. β-actin serves as the loading control. Note that the shRNA targets the 3'UTR of DMTF1. (**B & C**) Representative images (**B**) and quantification (**C**) of BrdU immunofluorescence of DMTF1 KD mouse NSCs, with or without overexpression of wildtype, ΔCDB or ΔMyb DMTF1. Scale bar: 20µm. (n=3 replicates, 6 fields/images per replicate) (mean ± SD). ns, not significant, ***P <0.001. Yellow arrows indicate BrdU^{+} cells.

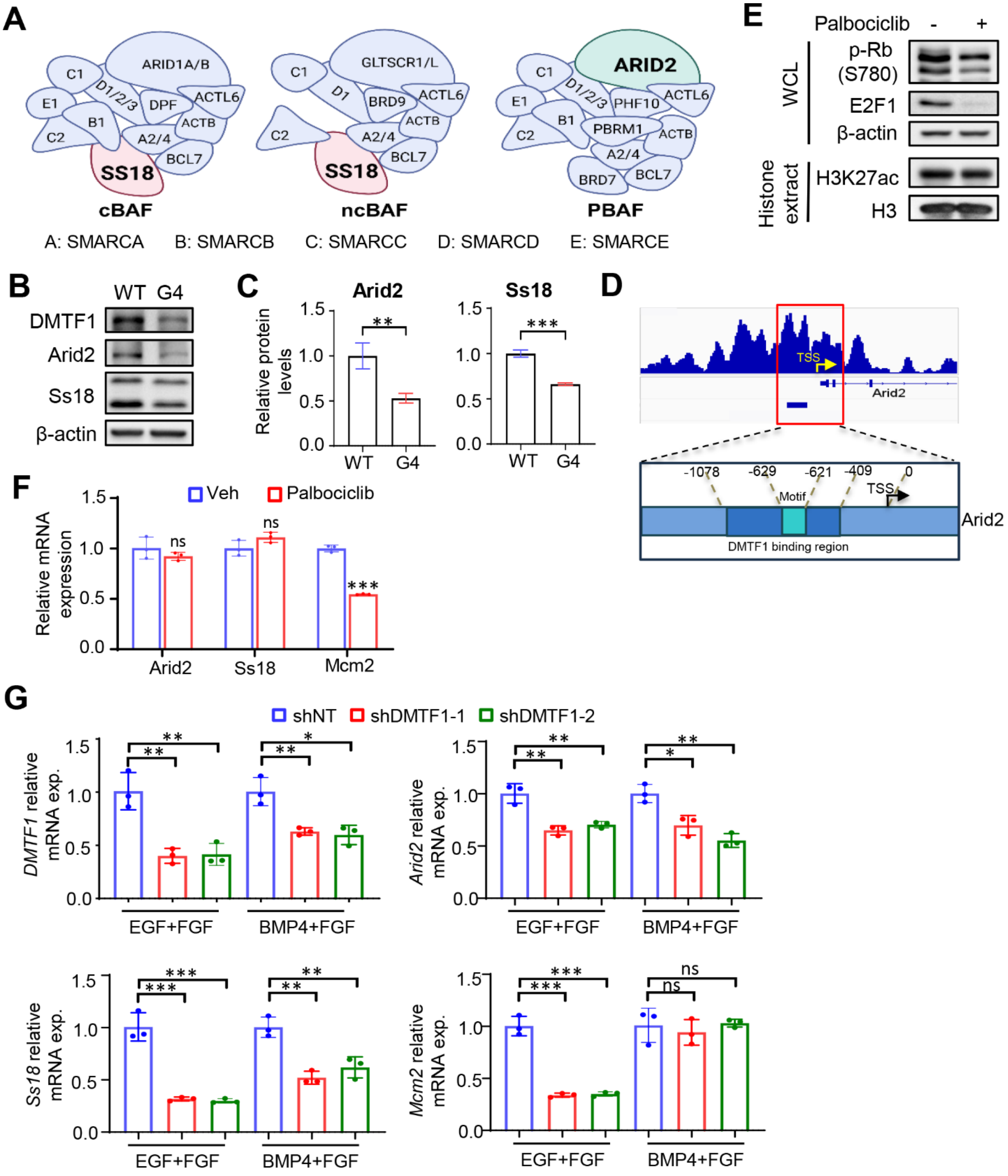

Fig. S5. Identification of Arid2 and Ss18 as bona fide DMTF1 gene targets in mouse NSC. (**A**) Schematic diagram of the three main types of SWI/SNF complexes. (**B**) Western blot analysis of DMTF1, Arid2 and Ss18 levels in WT and G4 TERT$^{ER/ER}$ mouse NSCs. β-actin serves as the loading control. (**C**) Quantification of Arid2 and Ss18 band intensity when normalized to loading control as shown below in (**B**) (n=3 replicates) (mean ± SD). $^{**}P$ <0.01, $^{***}P$ <0.001. (**D**) Schematic diagram showing the *Arid2* genomic region that contains the identified V5-DMTF1

ChIP-seq peak and motif. (**E**) Western blot analysis of phospho-Rb (p-Rb) and E2F1 levels in WCL, and H3K27ac levels in histone extracts of mouse NSCs that were treated with Palbociclib (10μM, 8hrs). β-actin serves as the loading control for WCL, while H3 serves as the loading control for histone extracts. (**F**) qPCR analysis of Arid2*,* Ss18 and MCM2 mRNA levels in mouse NSCs that are treated with Palbociclib (10μM, 8hrs). (n=3 replicates) (mean ± SD), ns, not significant, ***P <0.001. (**G**) qPCR analysis of DMTF1, Arid2, Ss18 and Mcm2 mRNA levels in DMTF1 KD mouse NSCs that are cultured with the indicated growth factors. (n=3 replicates) (mean ± SD), ns, not significant, *P <0.05, **P <0.01, ***P <0.001.

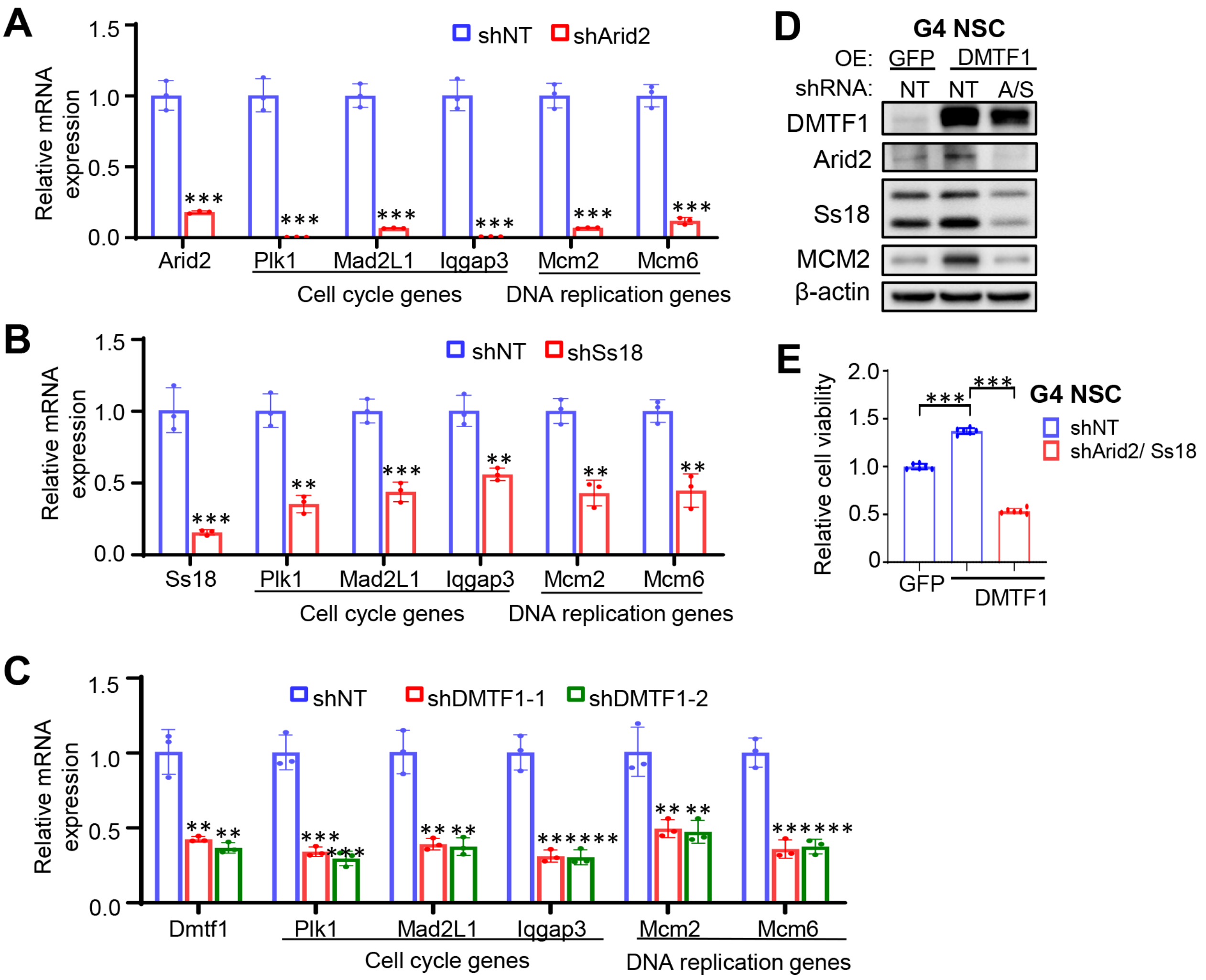

Fig. S6. Attenuated E2F1 program is likely mediated by Arid2 and Ss18 in DMTF1 KD mouse NSC. (**A-C**) qPCR analysis of representative cell cycle (Plk1, Mad2L1 and Iqgap3) and DNA replication genes (Mcm2 and Mcm6) in mouse NSC upon Arid2 (**A**), Ss18 (**B**) or DMTF1 (**C**) depletion. (n=3 replicates) (mean ± SD), **P <0.01, ***P <0.001. (**D**) Western blot analysis of DMTF1, Arid2, Ss18 and MCM2 levels in DMTF1 overexpressing G4 TERT$^{ER/ER}$ mouse NSCs, with or without Arid2 and Ss18 depletion. β-actin serves as the loading control. (**E**) Cell viability of DMTF1 overexpressing G4 TERT$^{ER/ER}$ mouse NSCs, with or without Arid2 and Ss18 depletion (n=6 replicates) (mean ± SD), ***P <0.001.

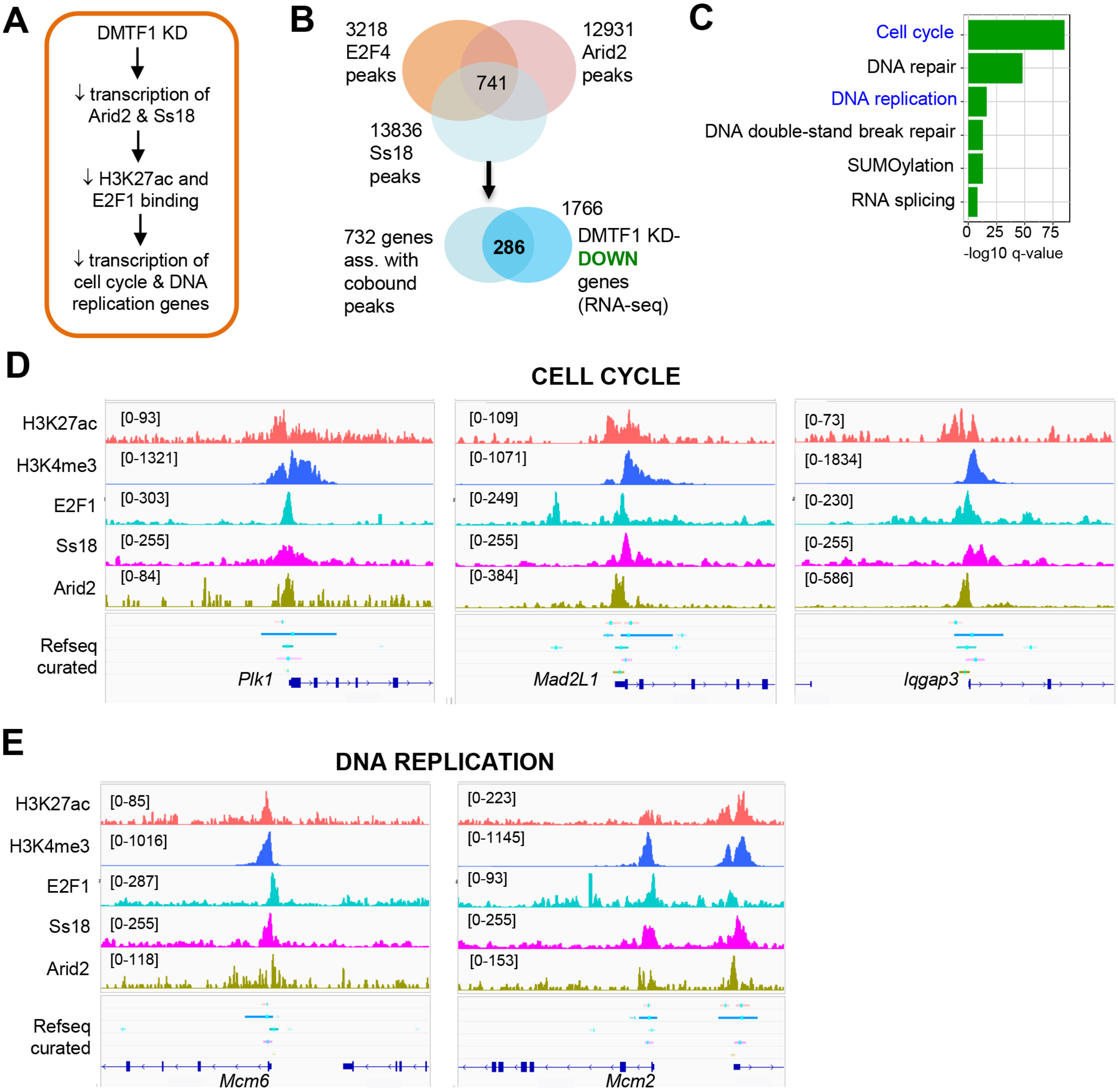

Fig. S7. Absence of DMTF1 reduces Arid2 and Ss18 occupancy at the promoter of E2F genes, leading to concomitant loss of gene activation and expression in mouse NSC. (**A**) Working model that may account for the molecular basis of DMTF1's role in NSC proliferation. (**B**) Venn diagram showing the number of downregulated genes in DMTF1 KD mouse NSC with Arid2/Ss18/E2F4 co-occupancy. (**C**) Pathway enrichment analysis of the 286 genes in (**B**). (**D & E**) Arid2, Ss18, E2F1, H3K27ac, and H3K4me3 ChIP-Seq tracks at the promoter of a subset of cell cycle (**D**) and DNA replication (**E**) genes.

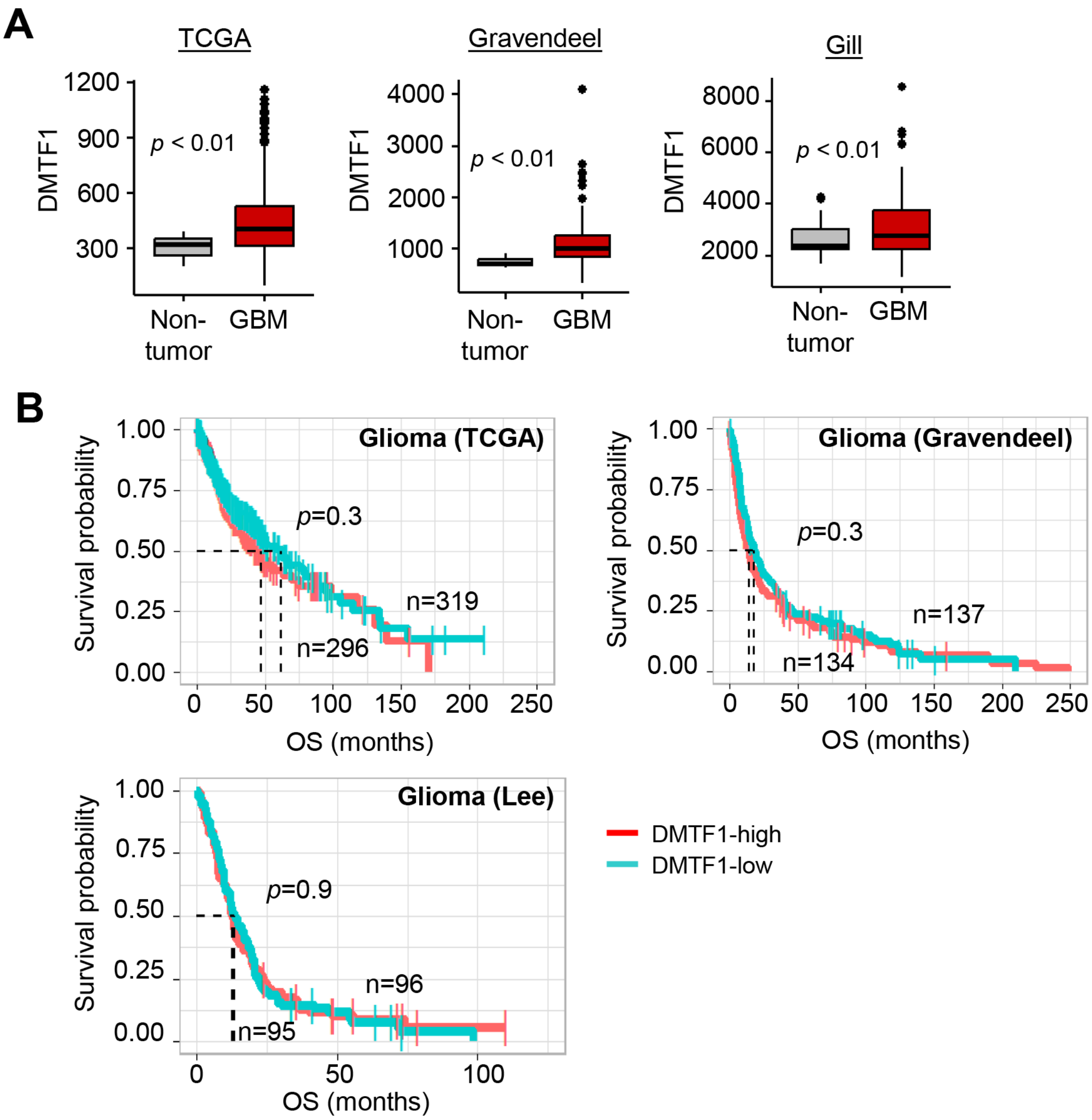

Fig. S8. DMTF1 expression does not inform unfavorable glioma patient outcome. (A) DMTF1 mRNA expression in non-tumor brain and GBM samples in three patient cohorts. Mann-Whitney test. **(B)** Kaplan–Meier survival analyses in three glioma patient cohorts. Median gene expression was used to split the $DMTF1^{low}$ and $DMTF1^{high}$ patient subgroups. Log-rank test.

Table S1. List of antibodies

**Antibodies**	**Brand**	**Cat number**
DMTF1 (human)	Novus	Cat# NBP1-84073
DMTF1 (mouse)	Invitrogen	Cat# PA5-29466
Arid2	Santa Cruz	Cat# sc-166117 X
Ss18	Cell Signaling	Cat# 21792S
Nestin (human)	Millipore	Cat# MAB5326
Nestin (mouse)	Millipore	Cat# MAB353
Sox2	Abcam	Cat# ab97959
Sox2	R&D Systems	Cat# MAB2018
MCM2	Abcam	Cat# ab4461
Ki67	Abcam	Cat# ab16667
BrdU	Biolegend	Cat# 339802
γH2ax	Millipore	Cat# 05-636
Cleaved caspase-3	Cell Signaling	Cat# 9661
ZO-1	Invitrogen	Cat# 61-7300
Myc-Tag	Cell Signaling	Cat# 9B11
V5-Tag (ChIP)	Thermo Fisher	Cat#A190-120A
V5-Tag (IF)	Cell Signaling	Cat# 13202
E2F1	Cell Signaling	Cat# 3742
E2F4	Santa Cruz	Cat# sc-398543
Foxm1	Cell Signaling	Cat#5605
c-Myc	Santa Cruz	Cat# sc-376471
p-Rb	Cell Signaling	Cat# 9307
p53(human)	Santa Cruz	Cat# sc-126
p53(mouse)	Cell Signaling	Cat# 32532
PCNA	Santa Cruz	Cat# sc-56
H3	Abcam	Cat# Ab1791

H3K27ac	Abcam	Cat# Ab4729
H2AZ	Active Motif	Cat# 39113
H3K4me3	Abcam	Cat# Ab8580
H3K9me3	Abcam	Cat# Ab8898
H3K27me3	Cell Signaling	Cat# 9733
Cdc6	Santa Cruz	Cat# sc9964
Orc2	Santa Cruz	Cat# 32734
Vinculin	Sigma-Aldrich	Cat# V9131
β-actin	Sigma-Aldrich	Cat# A5316
Goat anti-Mouse IgG Alexa Fluor™ 488	Invitrogen	Cat# A11001
Goat anti-Mouse IgG Alexa Fluor™ 555	Invitrogen	Cat# A32727
Goat anti-Mouse IgG Alexa Fluor™ 647	Invitrogen	Cat# A21235
Goat anti- Rabbit IgG Alexa Fluor™ 488	Invitrogen	Cat# A11008
Goat anti- Rabbit IgG Alexa Fluor™ Plus 555	Invitrogen	Cat# A32732
Goat anti- Rabbit IgG Alexa Fluor™ Plus 647	Invitrogen	Cat# A32733
HRP-Mouse	Cell Signaling	Cat# 7076S
HRP-Rabbit	Cell Signaling	Cat# 7074S

Table S2. sgRNA sequences used for CRISPR/Cas-9

**Name**	**Primer Sequence (5' › 3')**
DMTF1 sgRNA#1	GCAGCTGTGGCTGAACGAGT
DMTF1 sgRNA#2	GTAGTGTCCGTTCACCACAA

Table S3. Primer sequences for cloning

**Genes**	**Orientation**	**Primer Sequence (5' › 3')**
DMTF1- EcoRI	F	AAAGAATTCATGAGCACAGTGGAAGAGGATTCT
DMTF1- HindIII	R	AAAAAGCTTATGACAGTTTACCAAATCTTCGACATC

DMTF1- Gibson	F	AAGCGGCCCTGCAGATATCAGCCACCATGAGCACAGTGGAAGAGGATTCT
DMTF1- Gibson	R	GGGTTAGGGATAGGCTTACCATGACAGTTTACCAAATCTTCGACATCC
pHAGE- Gibson	F	GGTAAGCCTATCCCTAACCCTCTC
pHAGE- Gibson	R	TGATATCTGCAGGGCCGCTT
pHAGE-DMTF1$^{\Delta CDB}$-V5-GFP	F	GTGGGAAAATATACACCTG
pHAGE-DMTF1$^{\Delta CDB}$-V5-GFP	R	TTCAAAGCTCTGATCATTTTC
pHAGE-DMTF1$^{\Delta Myb}$-V5-GFP	F	GCAAACCATAAGGATGTTTC
pHAGE-DMTF1$^{\Delta Myb}$-V5-GFP	R	ATGGTTTCTGTCATCATAC
Ss18 promoter-NheI	F	AATGCTAGCCGAGACATTGCGGAAGGTTC
Ss18 promoter-HindIII	R	AATAAGCTTCTAGACTAGACGAGCGTGCA
Ss18 promoter mutant1	F	CGCCACGGCGGGAGGGAGCGG
Ss18 promoter mutant1	R	CCGCCCGATTTCCCCGCGGGC
Ss18 promoter mutant2	F	GCGTGATTTCGCCCTTG
Ss18 promoter mutant2	R	ATCCAGAAGGTGAGCC

Table S4. List of shRNAs used

Name	Primer Sequence (5’ › 3’)
shDMTF1#1 (mouse)	GCTGGTTGTAATTCAGCTTAT
shDMTF1#2 (mouse)	GCGATAATGTCACGGTACAAT
shDMTF1#1 (human)	TAAGGATTCTCTGACTAATAA
shDMTF1#2 (human)	CCACAATGGCTACGAAGTAAA
ShArid2	GACTAACAGCTGCCTTAATAT

ShSs18	GCTCGCAGTATCAGCAGATAT

Table S5. Primer sequences for ChIP-qPCR

**Genes**	**Orientation**	**Primer Sequence (5' › 3')**
Arid2	F	AGCTCCACAAAAGTAGGGCA
Arid2	R	CATGATGGGGCCGTAAACTC
Ss18	F	CTCTCCCGGCTCACCTTC
Ss18	R	CATGTCTGTGGCGTTCGC
Plk1	F	CGTAGTTGCTTCGTTCCTGG
Plk1	R	GCCGCCTTTAAACTCCGATC
Mad2L1	F	GTGGCGGGGAATAGCTTAGA
Mad2L1	R	CGCTCAGCACTCACAGAAAA
Iqgap3	F	GACGGGATTCCGCATTGAGA
Iqgap3	R	CCCTCCGGGTTCTGCATTTT
Mcm2	F	ACATGAGGAGAGCAGAGACG
Mcm2	R	CTTGGCCGGTATCTTCCTCT
Mcm6	F	AAGAAAGGAGTCGAGGAGGC
Mcm6	R	GCTGTTCCTAGACTTCCTGGA
NC-1	F	AGCTTCCAGGTGTTCTCTCC
NC-1	R	TCAATGGTACTGGACGTGCT
NC-2	F	TAAGAACAGAGAGGCCAGCC
NC-2	R	AGGCCTGTTACCCTTCACTC

Table S6. Primer sequences for qPCR

**Genes**	**Orientation**	**Primer Sequence (5' › 3')**
DMTF1	F	CCCAACGCTTTTGGAGAATA
DMTF1	R	GGACTGGAATCTGCAATGCT
Arid2	F	TGGATCTTCACGGGCTCTACA
Arid2	R	CGTTGGAACAACTTCTGGGAAA
Ss18	F	TTATCAGCAACCGTCGTATCCT

Ss18	R	GGGAGTTTCCTCCTTCGTAGTA
Plk1	F	CTTCGCCAAATGCTTCGAGAT
Plk1	R	TAGGCTGCGGTGAATTGAGAT
Mad2L1	F	GTGGCCGAGTTTTTCTCATTTG
Mad2L1	R	AGGTGAGTCCATATTTCTGCACT
Iqgap3	F	GTTGGATGGAGGTGTGCTTGA
Iqgap3	R	ACAGAGGGTGCAAAACAGTGG
Mcm2	F	ATCCACCACCGCTTCAAGAAC
Mcm2	R	TACCACCAAACTCTCACGGTT
Mcm6	F	ACCAACCCAAGGTTTGGAGG
Mcm6	R	TAATGCTCTCAGCGGTCTGTT
Gapdh	F	AGGTCGGTGTGAACGGATTTG
Gapdh	R	GGGGTCGTTGATGGCAACA
Actin	F	ACAGCCTGGATAGCAACG
Actin	R	CACCAACTGGGACGACAT
